# Monoamine oxidase A-dependent ROS formation modulates human cardiomyocyte differentiation through AKT and WNT activation

**DOI:** 10.1007/s00395-023-00977-4

**Published:** 2023-01-20

**Authors:** Moises Di Sante, Salvatore Antonucci, Laura Pontarollo, Ilaria Cappellaro, Francesca Segat, Soni Deshwal, Elisa Greotti, Luis F. Grilo, Roberta Menabò, Fabio Di Lisa, Nina Kaludercic

**Affiliations:** 1 Department of Biomedical Sciences, University of Padova, Via Ugo Bassi 58/B, 35131 Padua, Italy; 2 Max Planck Institute for Biology of Ageing, 50931 Cologne, Germany; 3 Neuroscience Institute, National Research Council of Italy (CNR), Via Ugo Bassi 58/B, 35131 Padua, Italy; 4 Center for Neuroscience and Cell Biology, University of Coimbra, 3004–504 Coimbra, Portugal; 5 Fondazione Istituto di Ricerca Pediatrica Città della Speranza (IRP), 35127 Padua, Italy

**Keywords:** Reactive oxygen species, hiPSCs, Cardiomyocyte differentiation, Development, Cell fate

## Abstract

During embryonic development, cardiomyocytes undergo differentiation and maturation, processes that are tightly regulated by tissue-specific signaling cascades. Although redox signaling pathways involved in cardiomyogenesis are established, the exact sources responsible for reactive oxygen species (ROS) formation remain elusive. The present study investigates whether ROS produced by the mitochondrial flavoenzyme monoamine oxidase A (MAO-A) play a role in cardiomyocyte differentiation from human induced pluripotent stem cells (hiPSCs). Wild type (WT) and MAO-A knock out (KO) hiPSCs were generated by CRISPR/Cas9 genome editing and subjected to cardiomyocyte differentiation. Mitochondrial ROS levels were lower in MAO-A KO compared to the WT cells throughout the differentiation process. MAO-A KO hiPSC-derived cardiomyocytes (hiPSC-CMs) displayed sarcomere disarray, reduced α- to β-myosin heavy chain ratio, GATA4 upregulation and lower macroautophagy levels. Functionally, genetic ablation of MAO-A negatively affected intracellular Ca^2+^ homeostasis in hiPSC-CMs. Mechanistically, MAO-A generated ROS contributed to the activation of AKT signaling that was considerably attenuated in KO cells. In addition, MAO-A ablation caused a reduction in WNT pathway gene expression consistent with its reported stimulation by ROS. As a result of *WNT* downregulation, expression of *MESP1* and *NKX2.5* was significantly decreased in MAO-A KO cells. Finally, MAO-A re-expression during differentiation rescued expression levels of cardiac transcription factors, contractile structure, and intracellular Ca^2+^ homeostasis. Taken together, these results suggest that MAO-A mediated ROS generation is necessary for the activation of AKT and WNT signaling pathways during cardiac lineage commitment and for the differentiation of fully functional human cardiomyocytes.

**Supplementary Information:**

The online version contains supplementary material available at 10.1007/s00395-023-00977-4.

## Introduction

Lineage specification is driven by a tight regulation of organ- and tissue-specific signaling cascades [56]. Among many factors, intracellular reactive oxygen species (ROS) play a crucial role during organogenesis, integrating cell redox state with differentiation [39, 71]. The nature of intracellular ROS and their spatiotemporal modulation is finely tuned and can regulate stem cell self-renewal, pluripotency, and differentiation [2, 45, 54]. Notably, H_2_O_2_ is directly involved in the physiological regulation of different signal transduction pathways by inducing post-translational modifications [23]. Mitochondrial ROS play a pivotal role during cardiac lineage commitment [11, 12], directly communicating with the cytosol, initiating and amplifying ROS-dependent signaling pathways that determine cell fate [21, 50, 76, 79]. Yet, mitochondrial enzymes responsible for ROS formation contributing to cardiomyogenesis remain unknown.

Mitochondrial electron transport chain is considered one of the main sites for mitochondrial ROS production [49]. However, other enzymes generate ROS within mitochondria [17]. In particular, monoamine oxidases (MAOs) are an important source of mitochondrial ROS in the heart and other tissues [1, 16, 25, 30, 32]. MAOs are a class of flavoenzymes that reside in the outer mitochondrial membrane and exist in two isoforms, MAO-A and B [4, 68], distinguished by different substrate specificity and inhibitor sensitivity. MAOs catalyze the degradation of endogenous monoamine neurotransmitters and biogenic amines relevant for cardiac function [32]. Importantly, the oxidative breakdown of substrates generates H_2_O_2_, thus representing a significant source of mitochondrial ROS [32]. The relative expression level of the two isoforms significantly differs in human tissues [60, 67]. Both enzymes are expressed in the heart, although MAO-A appears to be the predominant isoform [67]. The pathological role of MAOs in the heart has been extensively studied in the last decade. Excessive MAOs activation causes oxidative stress-mediated mitochondrial damage and cardiomyocyte necrosis [1, 16, 30, 33, 74], while lower/moderate levels of MAO-dependent H_2_O_2_ can trigger signaling cascades regulating cell growth [47]. Previous studies suggested a physiological role for MAO-A in embryonic brain development [75]. However, the involvement of MAO-dependent ROS as a mechanism underlying developmental processes has never been investigated.

Here, we tested the hypothesis that ROS deriving from MAOs directly participate in the regulation of human cardiac specification by activating ROS-dependent cardiomyogenic pathways. Specifically, we investigated whether (i) MAO-A is involved in mitochondrial ROS formation during human cardiomyocyte differentiation from human induced pluripotent stem cells (hiPSCs); (ii) MAO-A-dependent ROS generation can trigger the activation of ROS*-*sensitive signaling pathways that control fate commitment; (iii) MAO-A ablation affects cardiomyogenesis.

## Methods

A detailed, expanded Methods section is available in the Online Supplement.

### hiPSCs culture and treatments

hiPSCs (SCVI15) were kindly provided by Professor Joseph C. Wu (Stanford Cardiovascular Institute, USA). ATCC-BYS0112 hiPSCs were purchased by ATCC (ATCC, ACS-1026). hiPSCs line UST000013 was purchased from uSTEM s.r.l. Cells were maintained in Essential 8 Flex Medium (Thermo Fisher Scientific, USA) on Geltrex Matrix (Thermo Fisher Scientific) and passaged every 4 days using 0.5 mM ethylenediaminetetraacetic acid (EDTA) (Thermo Fisher Scientific) in Dulbecco’s PBS without Ca^2+^ or Mg^2+^ (Life Technologies). Cells were expanded in 6-well plates by passaging 1:8. Where indicated, MAO-A knock out (KO) cells were treated at day 4 of cardiac differentiation with 1 µM H_2_O_2_ for 2 h. To inhibit AKT activity, hiPSCs were incubated with 5 µM MK-2206 (SYNkinase) for 4 days during differentiation.

### Differentiation of hiPSCs into cardiomyocytes

hiPSCs were differentiated into cardiomyocytes using the STEMdiff™ cardiomyocyte differentiation kit (Stemcell Technologies, Canada) according to the manufacturer’s instructions. On day 14, beating cardiomyocytes were selected as described before [70]. hiPSCs-derived cardiomyocytes (hiPSC-CMs) were used for experiments 4 days after applying the selection protocol. The maturation of hiPSC-CMs was achieved by treating cells with maturation medium (MM) for 3 days as described before [20].

### Design and construction of CRISPRs targeting human MAO-A and lentiviral production

Lentiviral CRISPR/Cas9 expression constructs containing the guides for human MAO-A were generated using the lentiCRISPRv2 one vector system [59, 63]. Briefly, oligos were synthetized using Cas9 target design tool (ThermoFisher Scientific). Tested oligos are listed in Supplementary Methods. Lentiviral particles have been produced by transfecting HEK293T cells with four different plasmids [64]. To determine the efficacy of gene KO by lentiCRISPR transduction, single guide RNAs (sgRNAs) targeting MAO-A locus were tested in HEK293T cells (Supplementary Methods). EGFP sgRNA was used as control (Addgene, cat# 51,760) [63]. MAO-A and MAO-B expression levels were evaluated by western blot analysis after puromycin selection. Wells showing undetectable levels of MAO-A protein were chosen for knock-out cell line expansion and experiments.

### Live cell imaging

hiPSC-CMs were differentiated on µ-Plate 96-well black plates (Ibidi), experiments were carried out in an extracellular medium, images were acquired and quantified as previously described [1, 61].

To monitor mitochondrial ROS and mitochondrial membrane potential, cells were incubated with 25 nM MitoTracker Red CM-H_2_XRos, λ_exc_ 580 nm and λ_em_ 600 nm (MTR, Thermo Fisher Scientific), or 25 nM tetramethylrhodamine, λ_exc_ 550 nm and λ_em_ 580 nm (TMRM, Thermo Fisher Scientific), respectively. Images were collected at four different time points.

To monitor cytosolic Ca^2+^ transients, cells were loaded with Fluo-4 acetoxymethyl ester, λ_exc_ 490 nm and λ_em_ 515 nm (Thermo Fisher Scientific), 0.01% w/v pluronic F-127 (Sigma), and 250 µM sulfinpyrazone (Sigma). To evaluate the release of Ca^2+^ from the sarcoplasmic reticulum (SR), a pulse of 10 mM caffeine was added to the cells.

To monitor mitochondrial Ca^2+^, hiPSC-CMs were infected with an adenovirus containing mito-GCaMP5G [38] and 48 h later imaged both at baseline and following a pulse of 10 mM caffeine. Results are expressed as fluorescence intensity ratio F_λ1_/F_λ2_, in which λ_1_ (~ 410 nm) and λ_2_ (~ 480 nm), respectively.

### Mitophagy detection analysis

To monitor mitophagy levels in hiPSC-CMs, cells were plated on µ-Plate 96-well black plates (Ibidi) and the mitophagy detection Kit (Dojindo) was used. Briefly, cells were washed twice with PBS and incubated with 100 nmol/l Mtphagy Dye working solution at 37 °C for 30 min. Subsequently, the supernatant was discarded and wells washed twice with PBS. To induce mitophagy, positive control groups were incubated with FCCP (Sigma-Aldrich) 10 µM at 37 °C for 120 min. At the end of the induction time, medium was removed and cells washed twice with PBS. To observe the co-localization of Mtphagy Dye and lysosome, cardiomyocytes were incubated at 37 °C for 30 min with 1 μmol/l Lyso Dye working solution. Images were collected in live imaging solution (Thermo) using Crest X-light V3 confocal system and quantification of co-localization was determined using ImageJ FIJI. Three independent experiments were performed and at least ten fields of view per experiment were quantified.

### Citrate synthase activity assay

The citrate synthase activity in hiPSC-CMs was measured using the citrate synthase activity assay kit (Sigma-Aldrich) according to the manufacturer's instructions. Briefly, samples were diluted with citrate synthase assay buffer. After the dilution step, samples, positive controls and reduced glutathione (GSH) standard solutions were loaded into a 96-well plate. To start the reaction, 50 µL of mix containing citrate synthase developer and substrate were added to each well. Absorbance at 412 nm was measured every 5 min for 45 min at 25 °C. After background subtraction, citrate synthase activity was calculated according to the GSH amount (calculated using a standard curve), the reaction time and normalized to the total amount of protein loaded.

### Amplex red assay

Hydrogen peroxide formation was determined using Amplex Red assay as previously described [14]. Briefly, Amplex Red is a substrate of horseradish peroxidase (HRP), which in presence of H_2_O_2_ oxidizes 10-acetyl-3,7-dihyrdoxyphenoxazine (Amplex Red), resulting in the production of a red fluorescent compound resorufin (excitation/emission: 560/590 nm). hiPSCs were differentiated in 12-well plates and cells at D0, D2, and D4 were dissociated using StemPro™ Accutase™ Cell Dissociation Reagent (Thermo Fisher Scientific) for 5 min at room temperature. hiPSC-CMs (D20) were dissociated using Trypsin–EDTA 0.25% (Thermo Fisher Scientific) for 8 min at 37 °C. Cells were permeabilized with 50 µM digitonin (Sigma-Aldrich) in PBS for 3–4 min at 37 °C. Permeabilized cells were then incubated in PBS in the presence of 5 µM Amplex Red reagent and 4 µg/ml HRP, and dispensed into a black 96-well plate at different densities, ranging from of 2 × 10^5^ cells/well (D20) to 5 × 10^5^ cells/well (D0). The reaction was started adding 50 µM tyramine, and the resorufin fluorescence was monitored for 1 h at 37 °C using the Infinite 200 microplate plate reader (Tecan). Results are shown as pmol H_2_O_2_/min/million of cells. Since the production of H_2_O_2_ can derive from other cellular sources different than MAO-A, resorufin fluorescence was monitored in cells treated with 50 µM tyramine and 100 µM MAO inhibitor pargyline; the relative production of H_2_O_2_ was used as background and subtracted from the one induced by tyramine alone.

### Immunocytochemistry

Cells were prepared for staining using Cardiomyocyte Immunocytochemistry Kit (Thermo Fisher Scientific) following manufacturer’s instructions. Sarcomeres were stained using anti-α-sarcomeric actinin antibody (Sigma; 1:500, mouse) overnight at 4 °C. The day after, samples were incubated for 1 h at room temperature with Alexa Fluor 488 conjugated anti-mouse (Life Technologies, 1:250) and phalloidin TRITC conjugated (Sigma-Aldrich, 1:500) for actin staining. DAPI was used to stain nuclei (Invitrogen). Images were collected using Zeiss LSM 700 confocal system equipped with a PlanApo 40x/1.2 oil objective at 2048 × 2048 pixels per image with a 100 Hz acquisition rate, and analyzed as previously described [61].

### Western blot analysis

Cells were homogenized and protein concentration was determined using BCA protein assay (Pierce). Proteins were separated using SDS–PAGE (Invitrogen) and transferred to nitrocellulose membrane (Bio-Rad). Following incubation with primary and secondary HRP-conjugated antibodies (Bio-Rad), bands were detected and analyzed as previously described [1, 61]. Antibodies used in this study are listed in Supplementary Methods.

### Quantitative real time PCR analysis (qRT-PCR)

Total RNA was extracted using TRIzol (Invitrogen), and reverse transcription was performed using reverse SuperScript IV (Thermo Fisher Scientific). qRT-PCR was performed using Power SYBR Green PCR Master Mix (Applied Biosystems). Relative amounts of analyzed genes were calculated by the comparative ∆∆C(t) method. Primers used in this study are listed in Supplementary Methods.

### Data analysis

All values are expressed as mean ± S.E.M. Comparisons between groups were performed by either one-way or two-way ANOVA, followed by either Tukey’s or Dunn’s post hoc pairwise comparison when data were normally distributed. Data that did not follow normal distribution were analyzed by Kruskal–Wallis test, followed by Bonferroni post hoc multiple comparison. Comparisons between two groups were performed using two-tailed Student’s *t*-test. A value of *p* < 0.05 was considered significant.

## Results

### MAO-A is the only isoform expressed in hiPSCs and during main stages of cardiac differentiation

**Fig. 1:**
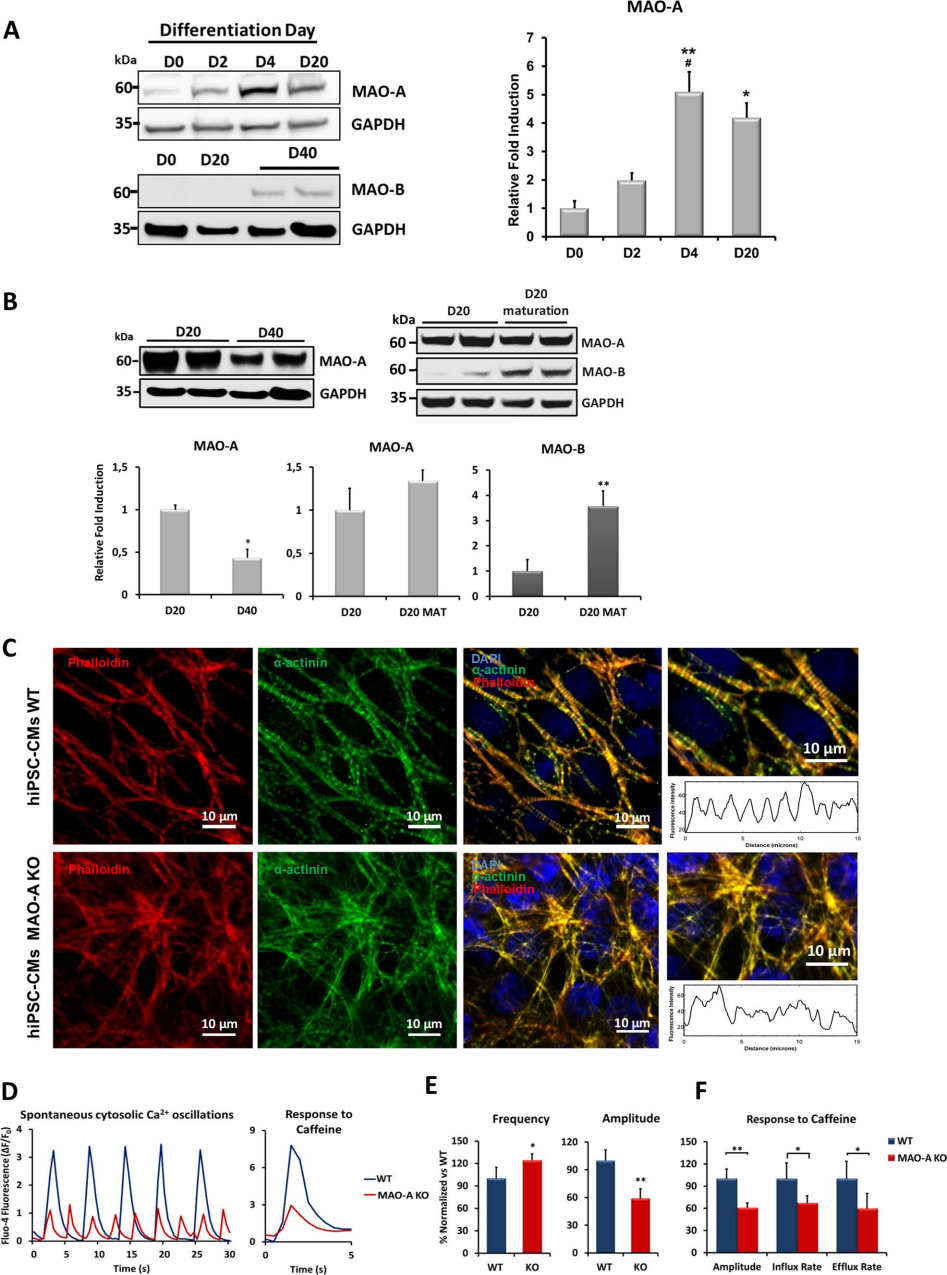
MAO-A and -B levels during cardiomyocyte differentiation and effect of MAO-A deletion on hiPSC-CMs structure and Ca^2+^ homeostasis. **A** MAO-A and MAO-B protein expression during different stages of cardiomyocyte differentiation. Densitometry analysis is shown on the right. MAO-A expression at day 0 (D0) in WT cells was arbitrarily considered as a unit. Values were normalized to GAPDH. *p < 0.05 vs D0, **p < 0.005 vs D0, #*p* < 0.05 vs D2 by one-way ANOVA, Dunn’s post hoc pairwise comparison. **B** MAO-A and MAO-B protein abundance during cardiomyocyte maturation. Densitometry analyses are shown in the lower panel. MAO-A or MAO-B expression at day 20 (D20) was arbitrarily considered as a unit. Values were normalized to GAPDH. *p < 0.05, **p < 0.01 by two-tailed Student’s *t* test. **C** Sarcomere organization in WT and MAO-A KO hiPSC-CMs assessed by immunofluorescent labeling of α-sarcomeric actinin (green). Phalloidin is shown in red, while nuclei were stained with DAPI (blue). The patterning of α-sarcomeric actinin fluorescence intensity is plotted on the right, denoting sarcomere organization within the cell. Approximately 20/30 cells were analyzed in each experiment. Scale bar 10 µm. **D** Representative traces of spontaneous cytosolic Ca^2+^ oscillations in WT and MAO-A KO hiPSC-CMs (left panel) and of cytosolic Ca^2+^ peak induced by caffeine stimulation (right panel). **E** Quantification of spontaneous cytosolic Ca^2+^ oscillations frequency and peak amplitude. *p < 0.05, **p < 0.001 by two-tailed Student’s *t* test. Five regions of interest (ROIs) were selected in each field of view and at least three different fields of view were analyzed in each experiment. **F** Quantification of cytosolic Ca^2+^ peak induced by caffeine stimulation in terms of peak amplitude, influx and efflux rate. *p < 0.05, **p < 0.001 by two-tailed Student’s *t* test. Kruskal–Wallis test was applied to not normally distributed data. All experiments were performed at least three times using three different preparations. Results are expressed as mean ± S.E.M

To investigate MAOs expression profile during human cardiomyogenesis, hiPSCs were differentiated into cardiomyocytes in vitro. During this process, MAO-A was the only isoform expressed and its expression increased during the first 20 days of cardiomyogenesis (Fig. 1A, Suppl. Figure 1A-B). The expression level of MAO-A was 5 times higher at the stage of mesoderm-cardiac specification (day 4) compared to undifferentiated cells (Fig. 1A). Importantly, the increase in MAO-A protein expression occurred independently from changes in mitochondrial mass (Suppl. Figure 1A). To rule out the possibility that these results were related to one specific genetic background, we assessed MAO-A expression levels in two additional hiPSCs lines obtained from healthy donors and subjected to differentiation. In accordance with the findings shown in Fig. 1A, the expression level of MAO-A protein increased during cardiomyocyte differentiation in a similar manner (Suppl. Figure 1B). On the contrary, MAO-B protein was undetectable during the entire process and became expressed after 20 days of culture, with a delayed but remarkable increase during the latter stages (day 40, Fig. 1A). Interestingly, MAO-A expression peaked at the mesoderm-cardiac specification and subsequently decreased over time by 40 days (Fig. 1B). Furthermore, when hiPSC-CMs were exposed to the MM to improve their metabolic maturation, MAO-B level significantly increased, whereas no major changes in MAO-A were detected (Fig. 1B).

These data suggest that MAO-A could be important during the early stages of cardiac commitment in developing human myocardium. Moreover, hiPSC-CMs represent a suitable in vitro model for the study of MAOs role during human cardiomyogenesis, since they express comparable levels of MAO isoforms found in the human heart [25].

### MAO-A ablation negatively affects hiPSC-CMs sarcomere structure and intracellular Ca^2+^ homeostasis leading to adaptive cardiomyocyte changes

To determine the role of MAO-A during cardiac specification, a loss-of-function approach was used to generate a stable isogenic MAO-A KO hiPSCs line by means of CRISPR/Cas9-mediated genome editing (Suppl. Figure 1C, D) [27]. MAO-A ablation did not impinge on the pluripotency potential, as revealed by the mRNA expression level of the pluripotency marker *NANOG* (Suppl. Figure 1E) and by immunostaining for NANOG and SSEA4 proteins (Suppl. Figure 1F). In both isogenic control and MAO-A KO cells the first spontaneously contracting cardiomyocyte clusters were observed in approximately 8–10 days. Noteworthy, the loss of the MAO-A isoform in hiPSCs and hiPSC-CMs was not accompanied by a compensatory expression of MAO-B (Suppl. Figure 1C, D).

WT hiPSC-CMs displayed a fully organized sarcomere structure after 20 days of differentiation, with a regular α-sarcomeric actinin striation pattern that was lost in MAO-A KO cells (Fig. 1C). To test if this structural alteration was paralleled by an impairment in Ca^2+^ homestasis, spontaneous cytosolic Ca^2+^ oscillations of WT and MAO-A KO hiPSC-CMs were monitored. The ablation of MAO-A led to a significant increase in Ca^2+^ oscillations frequency and a reduction in peak amplitude (Fig. 1D-E). In addition, the cytosolic Ca^2+^ peak induced by the release of Ca^2+^ from the sarcoplasmic reticulum (SR) following caffeine application, was reduced in mutant cardiomyocytes in terms of peak amplitude, influx and efflux rate (Fig. 1D and F). On the other hand, there were no significant differences in the mitochondrial Ca^2+^ content between WT and MAO-A KO hiPSC-CMs, either at baseline or after caffeine stimulation (Suppl. Figure 1G). Taken together, these data demonstrate that MAO-A ablation induces structural derangements and significant alterations in intracellular Ca^2+^ homeostasis in hiPSC-CMs.

**Fig. 2:**
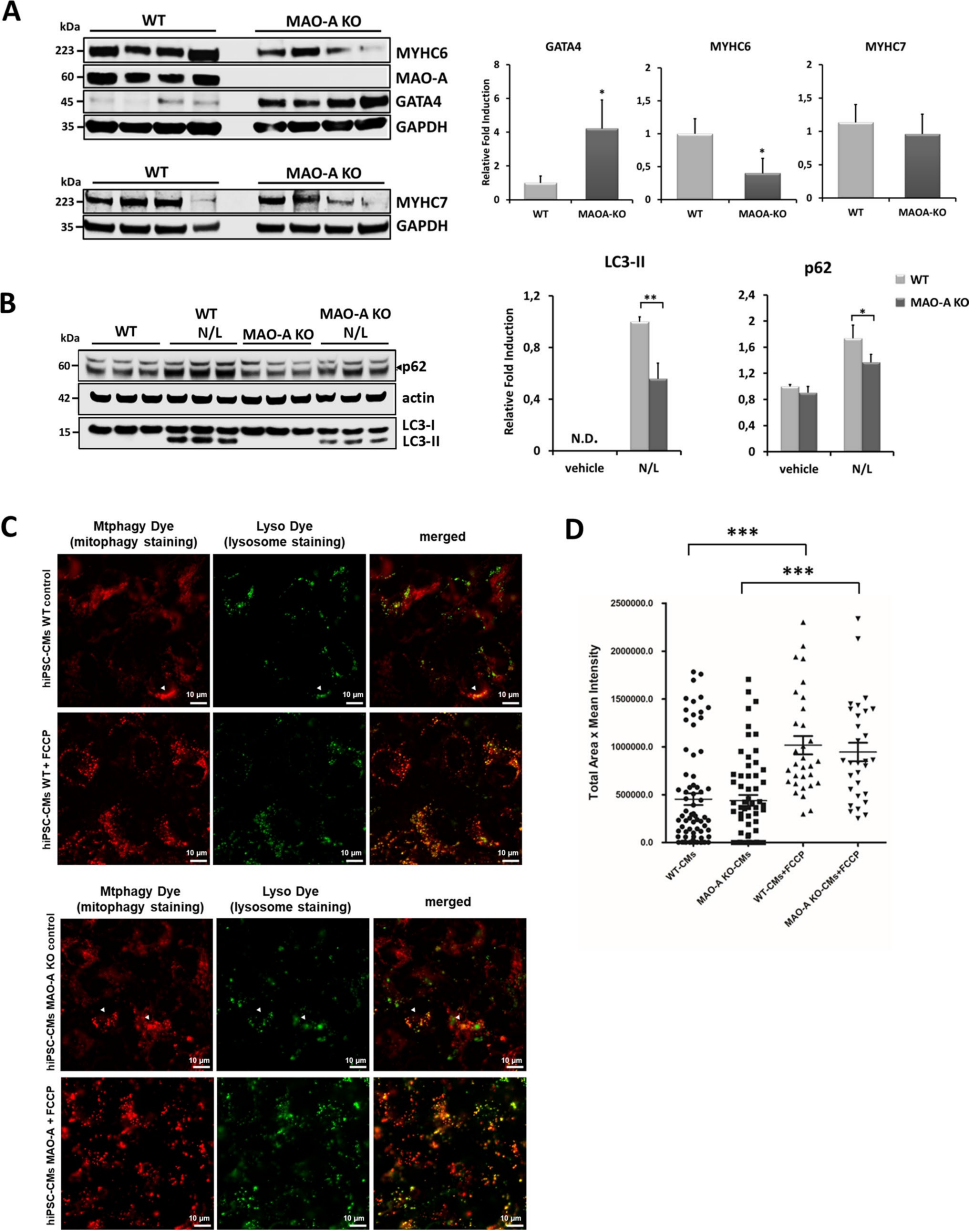
Effect of MAO-A deletion on cardiomyocyte-specific protein expression and autophagy/mitophagy in hiPSC-CMs. **A** MYHC6, MYHC7 and GATA4 protein levels in WT and MAO-A KO hiPSC-CMs. Densitometry analyses are shown on the right. Values were normalized to GAPDH. Protein levels in WT cells were arbitrarily considered as a unit. *p < 0.01 by two-tailed Student’s *t* test. **B** Representative western blots (left panel) and densitometry analyses (right panel) of p62, LC3B in WT and MAO-A KO hiPSC-CMs at baseline or after treatment with inhibitors of lysosomal degradation NH_4_Cl and leupeptin (N/L). Values were normalized to actin. LC3-II abundance in WT N/L was arbitrarily considered as a unit. p62 abundance in WT-vehicle was arbitrarily considered as a unit. *p < 0.05, **p < 0.001 by two-way ANOVA with post hoc Tukey’s multiple comparison test. **C** Representative images of Mtphagy Dye and Lyso Dye in hiPSC-CMs WT (upper panel) and MAO-A KO hiPSC-CMs (lower panel) stained cells in the absence or in the presence of FCCP; examples of co-localization regions are indicated by arrowheads. **D** Fluorescence intensity quantification in WT and MAO-A KO hiPSC-CMs at the basal level or with the induction of mitophagy by FCCP. Scale bar 10 µm. ***p < 0.001 by Kruskal–Wallis with post hoc Tukey’s multiple comparison test. All experiments were performed at least three times using three different preparations. Results are expressed as mean ± S.E.M

Deterioration of cardiac performance during cardiac remodeling arises from adaptive cardiomyocyte modifications, characterized by changes in gene expression [65], energy metabolism [28], sarcomeric protein composition [43], and autophagic response [34]. Several pathologic stimuli can cause a shift in the myosin heavy chain (MYHC) composition (i.e., MYHC6/MYHC7 ratio) [36, 48]. We thus explored the possibility that levels of these proteins could be altered in cells lacking MAO-A. MYHC6 levels were significantly reduced in MAO-A KO hiPSC-CMs, while no substantial differences were observed for MYHC7 (Fig. 2A). In addition, a marked increase in GATA4 was observed in MAO-A KO hiPSC-CMs when compared to their isogenic control (Fig. 2A), a condition that resembles data obtained in dysfunctional heart [53]. On the other hand, proteins involved in the regulation of Ca^2+^ homeostasis were unaltered at the transcriptional level between WT and MAO-A KO hiPSC-CMs (Suppl. Figure 2A). In addition, the phosphorylation status of phospholamban (PLN) appeared unchanged (Suppl. Figure 2B), indicating that in MAO-KO hiPSC-CMs the regulation of SERCA2A by PLN is preserved. To assess whether the genetic ablation of an outer mitochondrial membrane protein (i.e., MAO-A) could have potentially altered the interaction between mitochondria and endoplasmic/sarcoplasmic reticulum (ER/SR), we examined subcellular structures in MAO-A KO cells by means of transmission electron microscopy (TEM). The distance between ER and mitochondria the ER–mitochondria contact coefficient (ERMICC) appeared unchanged in WT and MAO-A KO hiPSCs or hiPSC-CMs (Suppl. Figure 2C–D).

Alterations in autophagy levels in the heart frequently occur in response to stress [31]. Here we tested whether structural and functional alterations observed in MAO-A KO hiPSC-CMs could be accompanied by an alteration of autophagy flux. Interestingly, macroautophagy levels were reduced in MAO-A KO hiPSC-CMs, as evidenced by reduced accumulation of LC3B-II and p62 in the presence of inhibitors of lysosomal degradation (i.e., leupeptin/NH_4_Cl) (Fig. 2B). However, mitophagy levels remained unchanged in MAO-A-KO hiPSC-CMs either at basal level or after the induction of mitophagy (Fig. 2. C–D). In line with this evidence, mitochondrial content in cardiomyocytes was equally balanced in both cell types (Suppl. Figure 2E) confirming that the mitochondrial mass was not affected.

### MAO-dependent ROS generation contributes substantially to mitochondrial ROS levels during cardiac commitment

**Fig. 3:**
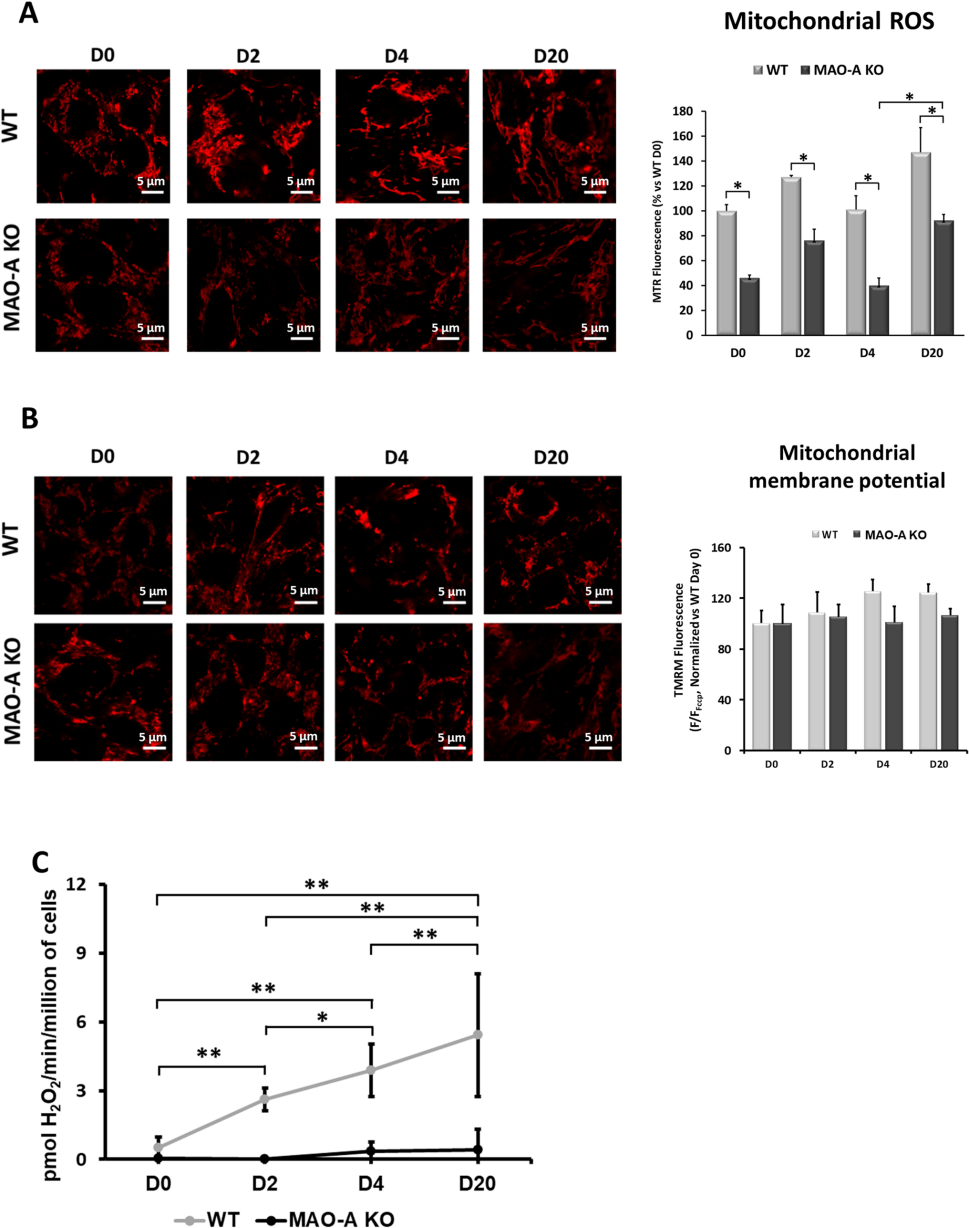
Effect of MAO-A deletion on mitochondrial ROS formation during cardiomyocyte differentiation. **A** Representative images of MitoTracker Red CM-H_2_XRos (MTR) stained cells (left panel) and fluorescence intensity quantification (right panel) in WT and MAO-A KO cells throughout different stages of differentiation. Values were normalized and expressed as % vs WT D0. *p < 0.05, by two-way ANOVA with post hoc Tukey’s multiple comparison test. At least 100 cells were analyzed per condition in each experiment. Scale bar 5 µm. **B** Mitochondrial membrane potential in WT and MAO-A KO cells at day 0 (D0), day 2 (D2), day 4 (D4) and day 20 (D20). Quantification of TMRM fluorescence intensity evaluated before and after the addition of FCCP is shown on the right. Results are expressed as F/F_FCCP_, normalized to WT D0 and statistically analyzed by two-way ANOVA. At least 30 cells were analyzed per condition in each experiment. Scale bar 5 µm. **C** MAO activity was measured fluorometrically in permeabilized cells at different time points during cardiac commitment (D0, D2, D4 and D20). Amplex Red fluorescence was measured kinetically after the administration of the MAOs substrate tyramine. Data were compared to MAO activity at D0 and statistically analyzed by Kruskal–Wallis with post hoc Bonferroni test. All experiments were performed at least three times using three different preparations. Results are expressed as mean ± S.E.M

A constant rise in mitochondrial H_2_O_2_ formation has been observed during cardiac commitment, from hiPSCs to hiPSC-CMs [44]. We examined to what extent MAO-A generated H_2_O_2_ was contributing to mitochondrial ROS levels during this process. MAO-A KO hiPSCs displayed remarkably lower levels of mitochondrial ROS in all stages compared to their isogenic control (Fig. 3A) whereas mitochondrial membrane potential remained unaltered (Fig. 3B), suggesting that MAO-A is prominently involved in mitochondrial ROS generation throughout the differentiation process. To better correlate MAO-A expression and enzyme activity during differentiation, we measured MAO-A dependent ROS formation during different stages of differentiation in the presence of exogenously added MAO substrate tyramine. Results shown in Fig. 3C show that there is a progressive increase in ROS formation over time in WT cells that reaches a peak at days 4 and 20, in accordance with the increase in MAO-A protein expression over time. This indicates that the potential for MAO-dependent ROS formation increases during cardiomyocyte differentiation and depends on the substrate availability. Moreover, considering that MAO-B genetic locus is still functional in MAO-A deleted cells, this result suggests that there is no compensatory effect elicited by MAO-B activity.

To test for potential consequences of indiscriminate ROS scavenging on human cardiomyocyte differentiation, hiPSCs were differentiated in the presence of either MAO-A inhibitor pargyline, a general ROS scavenger N-2-mercaptopropionylglycine (MPG), or a specific mitochondrial ROS buffer (mitoTEMPO). Either mitochondrial or total ROS scavenging severely impaired cardiac differentiation, with null or few spare contracting foci observed in treated wells (Suppl. Figure 3A) [40]. In line with our previous results (Fig. 1 C-F), treatment with MAO inhibitor pargyline did not block the formation of beating foci (Suppl. Figure 3A) and it partially reduced *NKX2.5* gene expression (Suppl. Figure 3B). This supports the evidence that, although impaired, differentiation of beating cardiomyocytes is still able to occur when MAO-A is downregulated/inhibited. In contrast, mitochondrial ROS scavenging with mitoTEMPO was sufficient to impair cardiac differentiation, as demonstrated by the drastic reduction of *NKX2.5* gene expression, a marker of cardiac commitment (Suppl. Figure 3B). Altogether, these results confirm the pivotal role of ROS during cardiac differentiation process and highlight the role of MAO-A generated ROS in cardiac lineage commitment.

### MAO-A dependent ROS formation modulates AKT and WNT signaling pathways

**Fig. 4:**
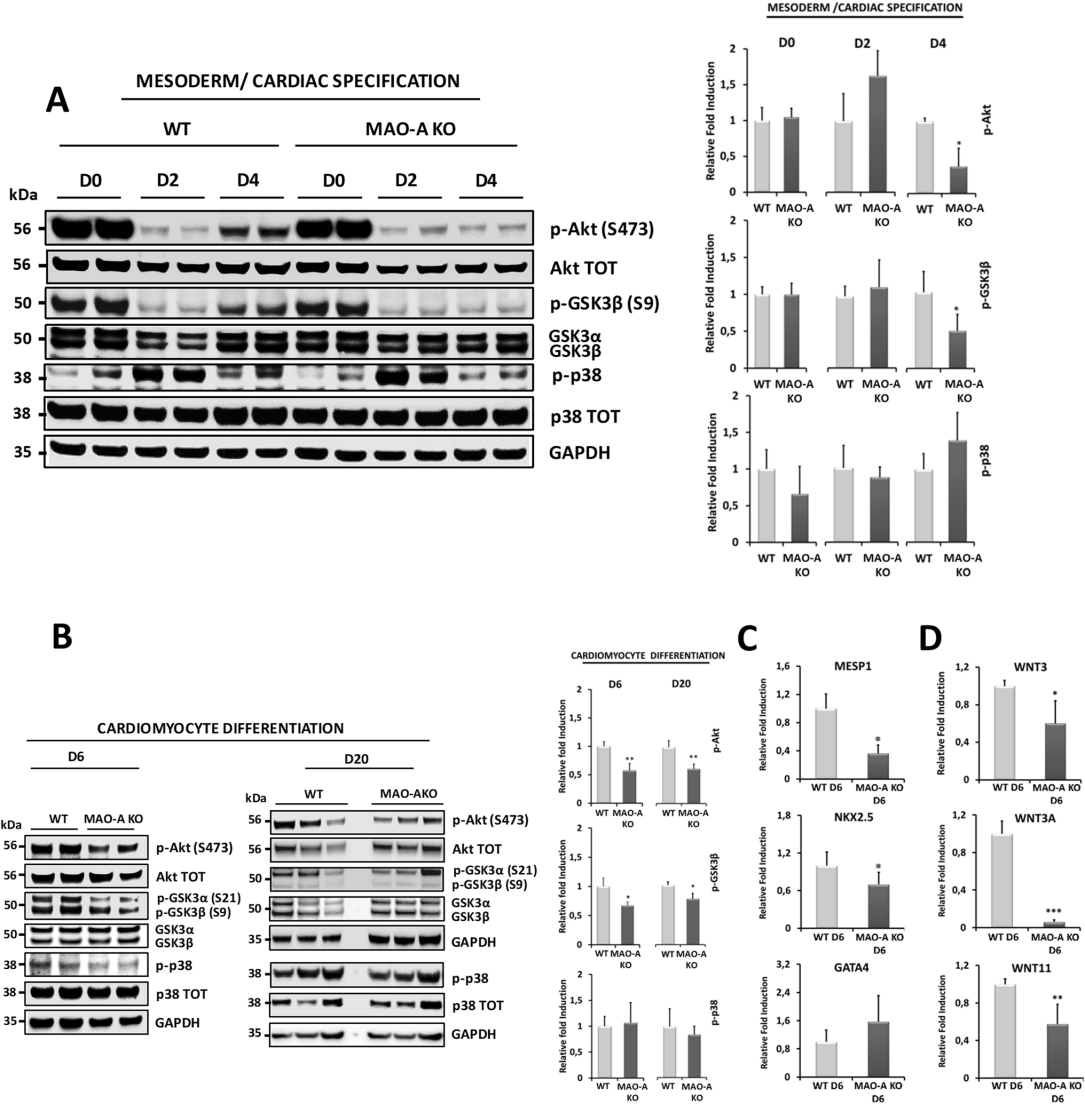
Effect of MAO-A deletion on AKT and WNT signaling pathways during cardiomyocyte differentiation. **A** AKT, GSK3β and p38 phosphorylation was assessed at day 0 (D0), day 2 (D2) and day 4 (D4) during mesoderm/cardiac specification in WT and MAO-A KO cells. Densitometry analyses are shown on the right. For each group, WT values were arbitrarily considered as a unit. Phosphorylation levels were normalized to total protein levels. *p < 0.001 by two-tailed Student’s *t* test. **B** AKT, GSK3β and p38 phosphorylation was assessed in WT and MAO-A KO cells at day 6 (D6) and day 20 (D20) during cardiomyocyte differentiation. Densitometry analyses are shown on the right. For each group, WT values were arbitrarily considered as a unit. Phosphorylation levels were normalized to total protein levels. *p < 0.05, **p < 0.01 by two-tailed Student’s *t* test. **C** *MESP1*, *NKX2.5*, and *GATA4* mRNA expression levels in WT and MAO-A KO cells assessed at day 6 (D6). For each gene, WT values were arbitrarily considered as a unit. Values were normalized to *GAPDH*. *p < 0.01 by two-tailed Student’s *t* test. **D** *WNT3*, *WNT3A* and *WNT11* mRNA expression levels in WT and MAO-A KO cells assessed at day 6 (D6). For each gene, WT values were arbitrarily considered as a unit. Values were normalized to *GAPDH*. *p < 0.05, **p < 0.01, ***p < 0.001 by two-tailed Student’s *t* test. All experiments were performed at least three times using three different preparations. Results are expressed as mean ± S.E.M

Next, we tested whether MAO-A ablation impairs differentiation by altering ROS-dependent signaling pathways. AKT and p38 MAPKs, master regulators of cardiac lineage commitment, can be modulated by ROS [3, 35, 51, 55, 78]. Thus, we sought to determine whether differences in AKT and p38 phosphorylation/activation between WT and MAO-A KO cells were detectable during mesoderm/cardiac specification (from days 1 to 4) and during cardiac differentiation (from days 6–20). AKT phosphorylation showed a biphasic response during the initial stages of cardiac specification and was significantly reduced in MAO-A KO vs WT cells at day 4 (Fig. 4A). A reduction in phosphorylation levels of GSK3β at serine 9 (S9) was also observed in MAO-A KO cells (Fig. 4A), indicating an overall decrease of the AKT signaling cascade. In addition, a reduction in AKT and GSK3β phosphorylation persisted during formation of cardiac precursors (day 6) and in beating cardiomyocytes (day 20, Fig. 4B). Conversely, no significant alterations in p38 MAPK phosphorylation were detected in MAO-A KO hiPSC-CMs (Fig. 4A–B). Of note, we found that phosphatase *INPP4A* was significantly upregulated in MAO-A KO hiPSC-CMs (Suppl. Figure 3C). INPP4A is a lipid phosphatase that dephosphorylates PtdIns(3,4)P_2_ to form PtdIns(4)P and PtdIns(3)P, acting as a negative regulator of the PI3K/AKT pathway. Recently, we showed that MAO activity is able to regulate miR-27a-3p levels that, in turn, binds *Inpp4a* mRNA and modulates its levels [10]. Taken together, these findings suggest that the activity of INPP4A is likely increased in MAO-A KO cells, thereby resulting in impaired signal transduction and AKT activation.

To further corroborate the significance of these findings, we tested whether dysregulation of the AKT/GSK3β pathway in MAO-A KO cells could alter the expression of genes related to myocardial commitment such as *MESP1*, the primary cardiac mesoderm regulator, *GATA4* and *NKX2.5*, two key factors required for cardiac specification. MAO-A KO cells displayed a significant down regulation of both *MESP1* and *NKX2.5* genes at day 6, but no statistically significant changes in *GATA4* levels have been detected (Fig. 4C). Activation of the AKT cascade alone is necessary but not sufficient to support cardiac differentiation. Indeed, this process involves also WNT pathway that with AKT signaling converges in causing GSK3β inactivation [51]. Temporally controlled canonical/non-canonical WNT axes are strongly implicated in the specification of mesoderm cells toward cardiac progenitors and subsequent cardiomyocyte formation [37, 42]. Notably, ROS have been reported to stimulate the WNT pathway through the dissociation of disheveled from oxidized nucleoredoxin [24]. Interestingly, ROS appear to be linked to WNT in an amplification pathway whereby stimulation of NOX1 activity downstream of the WNT receptor causes an increase in ROS levels that eventually promote WNT-related gene expression [18, 29]. In our experimental model, pharmacological inhibition of NOX activity with apocynin or treatment of the cells with Wnt-C59 (a potent WNT signaling pathway inhibitor) during cardiac differentiation drastically reduced the percentage of beating foci (Suppl. Figure 3D). On the other hand, mitochondrial ROS formation has rarely been considered [57, 73]. A correlation between MAO inhibition and WNT signaling regulation was explored in the treatment of Alzheimer’s disease with a new hybrid compound able to inhibit both MAO and cholinesterase [58]. In addition, MAO inhibition has been proposed to control the proliferative potential of lymphoma cells [77]. However, no information is available on MAO-induced ROS formation and WNT signaling in the context of cardiomyocyte lineage commitment. Expression level of key genes belonging to the *WNT* family was unchanged between WT and KO cells at day 4 (Suppl. Figure 3E). Nevertheless, a drastic downregulation in *WNT3A* gene expression was observed in KO cells at day 6, with concomitant reduction in *WNT3* and *WNT11* gene expression levels (Fig. 4D). Of note, mitochondrial ROS levels were reduced in MAO-A KO cells also at day 6, while mitochondrial membrane potential remained unaffected (Suppl. Figure 3F-G).

To rule out a possible involvement of AKT in *WNT* gene expression regulation during cardiac differentiation of hiPSCs, we used a highly selective AKT inhibitor MK-2206 (Suppl. Figure 3H) during the first 4 days of differentiation and measured the expression level of *WNT3*, *WNT3A* and *NKX2.5* genes at day 6. Pharmacological AKT inhibition reduced *NKX2.5* expression level thereby confirming its role in cardiomyogenesis [51], but it did not affect *WNT3* or *WNT3A* expression (Suppl. Figure 3I), suggesting that transcription of these genes is not directly controlled by AKT activity.

Taken together, our data show that persistent alteration of the mitochondrial redox balance in MAO-A KO cells negatively affects AKT/GSK3β pathway at day 4 and *WNT* axis later on. Impaired activity of these signaling cascades in MAO-A KO cells causes a reduction in the expression of cardiac transcription factors that impinges on cardiomyocyte commitment.

### Re-expression of MAO-A during lineage commitment restores AKT/GSK3β and WNT pathways signaling and partially rescues phenotype alterations

**Fig. 5:**
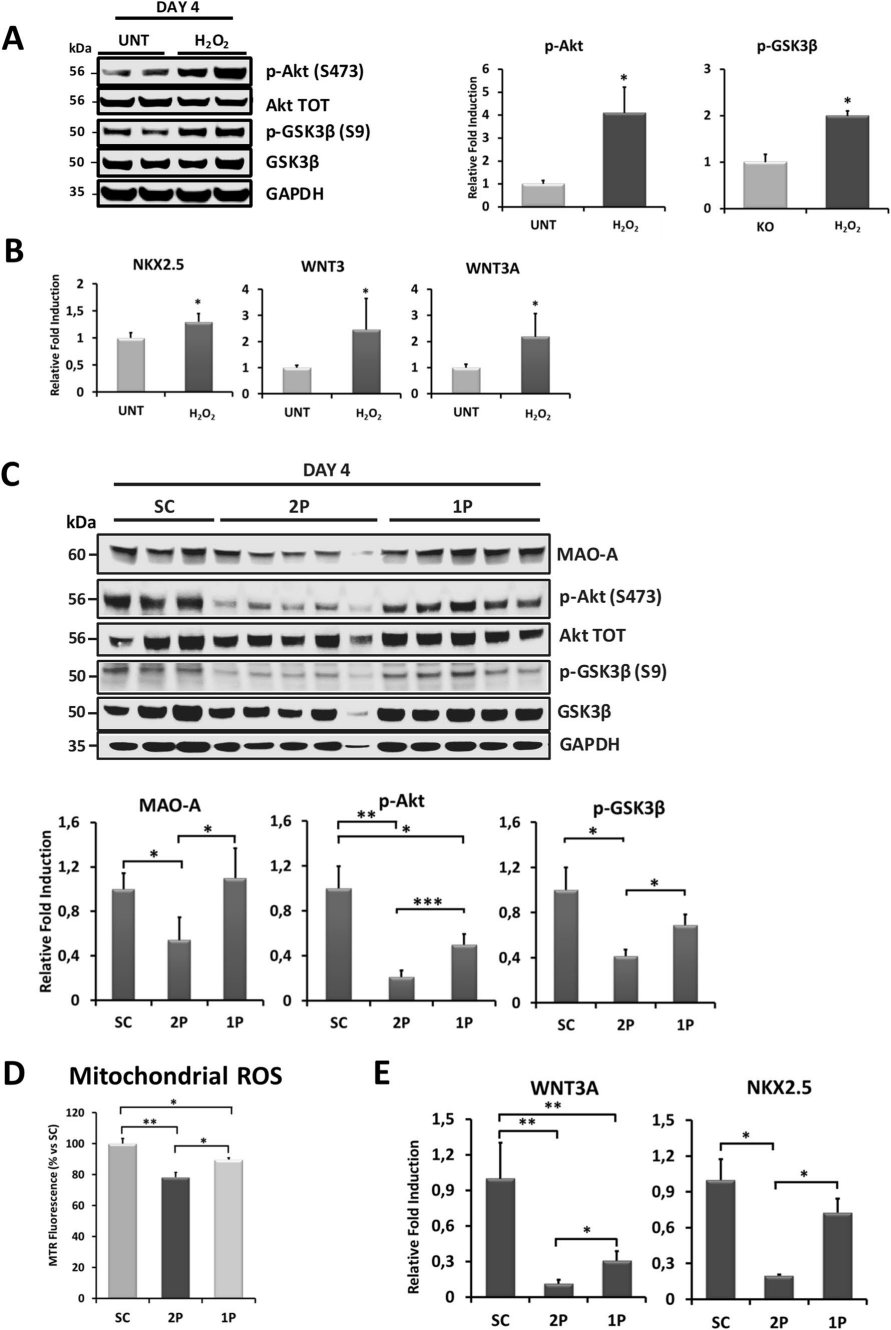
Effect of H_2_O_2_ bolus treatment and MAO-A re-expression on AKT and WNT pathways during cardiac differentiation. **A** AKT and GSK3β phosphorylation following a bolus addition of H_2_O_2_ to MAO-A KO cells at day 4 (D4) of cardiac differentiation. Densitometry analyses are shown on the right. AKT and GSK3β phosphorylation levels in MAO-A KO untreated (UNT) cells were arbitrarily considered as a unit. Phosphorylation levels were normalized to total protein levels. *p < 0.01 by two-tailed Student’s t test. **B** *NKX2.5*, *WNT3* and *WNT3A* mRNA expression level in MAO-A KO cells at day 6 following H_2_O_2_ bolus treatment. For each gene, MAO-A KO untreated (UNT) values were arbitrarily considered as a unit. Values were normalized to *GAPDH*. *p < 0.05 by two-tailed Student’s *t* test. **C** MAO-A protein levels and AKT/GSK3β phosphorylation following treatment with scramble (SC) RNA, 1 pulse (1P) or 2 pulses (2P) of siRNA against MAO-A. Densitometry analyses for MAO-A expression levels, and AKT and GSK3β phosphorylation at day 4 are shown in the lower panel. For each group, SC values were arbitrarily considered as a unit. Phosphorylation levels were normalized to total protein levels. *p < 0.05, **p < 0.01, ***p < 0.001 by one-way ANOVA with post hoc Tukey’s multiple comparison test. **D** Mitochondrial ROS levels at day 4 following treatment with scramble (SC) RNA, 1 pulse (1P) or 2 pulses (2P) of siRNA against MAO-A. Values were normalized and expressed as % vs SC. *p < 0.01, **p < 0.001 by one-way ANOVA with post hoc Tukey’s multiple comparison test. At least 100 cells were analyzed per condition in each experiment. **E** *WNT3A* and *NKX2.5* mRNA expression levels at day 6 in cells treated with scramble (SC) RNA, 1 pulse (1P) or 2 pulses (2P) of MAO-A siRNA. For each gene, SC values were arbitrarily considered as a unit. Values were normalized to *GAPDH*. *p < 0.05, **p < 0.01 by one-way ANOVA with post hoc Tukey’s multiple comparison test. All experiments were performed at least three times using three different preparations. Results are expressed as mean ± S.E.M

To further corroborate the hypothesis that cells are particularly sensitive to ROS oscillations at day 4 of differentiation, we exposed MAO-A KO cells to a pulse of H_2_O_2_ at that time point. A significant increase in AKT and GSK3β phosphorylation was observed after 2 h (Fig. 5A). In addition, this single pulse of H_2_O_2_ led to an increase in *NKX2.5*, *WNT3* and *WNT3A* gene expression at day 6 (Fig. 5B). Conversely, treatment of WT cells with mitoTEMPO for 4 days drastically reduced the expression level of *WNT3A* at day 6 (Suppl. Figure 3 J). This *WNT3A* reduction upon mitoTEMPO treatment is in line with the results obtained for *NKX2.5* gene expression (Suppl. Figure 3B), confirming that buffering mitochondrial ROS impinges on cardiac differentiation.

Finally, we hypothesized that reactivation of MAO-A at day 4 could restore the activity of AKT and WNT pathways, rescuing the altered phenotype observed in MAO-A KO hiPSC-CMs. Given that MAO-A expression is tightly regulated during cardiac commitment (Fig. 1A) and its excessive activation results in oxidative stress [32], we took advantage of the siRNA strategy to transiently reduce MAO-A protein levels only in the first phase of differentiation (Suppl. Figure 4A). Cells were treated with MAO-A siRNA at two different time points (2 pulses (2P) protocol), specifically at the stage of hiPSCs (1^st^ pulse, day 0) and between mesoderm and cardiac specification (2nd pulse, day 2), leading to the generation of MAO-A knock down (KD) cells that display 50% reduction in MAO-A protein level at days 2 and 4 (Suppl. Figure 4B, Fig. 5C). In parallel, a single siRNA administration was used to silence MAO-A only in the first 2 days of differentiation (1 pulse (1P) protocol, day 0, Suppl. Figure 4A–B), allowing the cells to naturally re-express MAO-A to the physiological levels by day 4 (Fig. 5C). Regardless of the protocol used, MAO-A protein levels were restored by day 6 (Suppl. Figure 4C). Similarly to MAO-A KO hiPSC-CMs (Fig. 4A), MAO-A KD by the 2P protocol significantly reduced AKT and GSK3β phosphorylation (Fig. 5C). Notably, MAO-A re-expression by means of 1P protocol partially but significantly restored AKT and GSK3β phosphorylation (Fig. 5C). This result was paralleled by the restoration of mitochondrial ROS levels at day 4 and *WNT3A* and *NKX2.5* expression at day 6 in 1P- compared to 2P-treated cells (Fig. 5D–E).

**Fig. 6:**
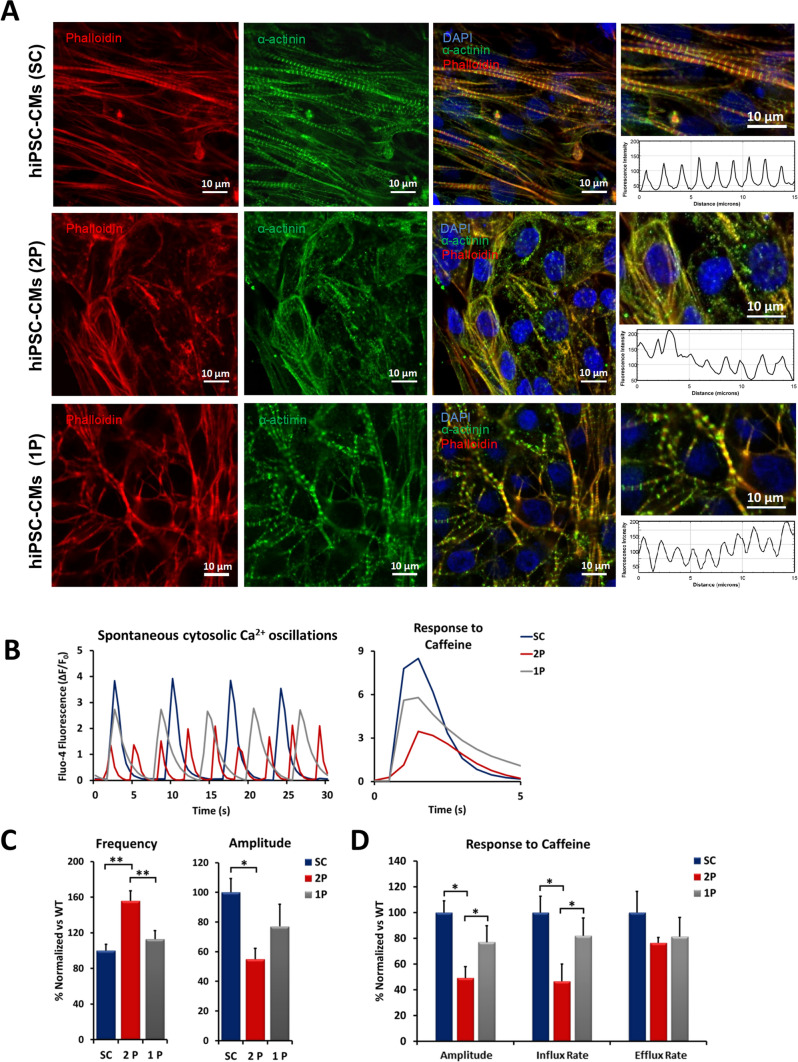
Effect of MAO-A re-expression on hiPSC-CMs sarcomere organization and Ca^2+^ homeostasis. **A** α-sarcomeric actinin (green) immunofluorescent labeling in cells treated with scramble (SC) RNA, 1 pulse (1P) or 2 pulses (2P) of MAO-A siRNA. Phalloidin is shown in red, while nuclei were stained with DAPI (blue). The patterning of α-sarcomeric actinin fluorescence intensity is plotted on the right, denoting sarcomere organization within the cell. Approximately 20/30 cells were analyzed in each experiment. Scale bar 10 µm. **B** Representative traces of spontaneous cytosolic Ca^2+^ oscillations in scramble (SC) RNA, 1 pulse (1P) or 2 pulse (2P) MAO-A siRNA treated hiPSC-CMs (left panel) and of cytosolic Ca^2+^ peak induced by caffeine stimulation (right panel). **C** Quantification of spontaneous cytosolic Ca^2+^ oscillations frequency and peak amplitude. *p < 0.01, **p < 0.001 by one-way ANOVA with post hoc Tukey’s multiple comparison test. Five regions of interest (ROIs) were selected in each field of view and at least three different fields of view were analyzed in each experiment. **D** Quantification of caffeine-induced cytosolic Ca^2+^ peak in terms of peak amplitude, influx and efflux rate. *p < 0.01 by one-way ANOVA with post hoc Tukey’s multiple comparison test. Where data were not normally distributed, Kruskal–Wallis test was applied. All experiments were performed at least three times using three different preparations. Results are expressed as mean ± S.E.M

In line with results obtained in MAO-A KO cells, the majority of cardiomyocytes derived from hiPSCs subjected to the 2P protocol exhibited myofilament disarray that was not detected in cells treated with 1P protocol (Fig. 6A). Moreover, 2P MAO-A KD cells displayed a significant increase in the frequency of spontaneous Ca^2+^ oscillations in the cytosol and a significant decrease in the peak amplitude (Fig. 6B–C). The 2P protocol induced an impairment in response to caffeine in KD cells, with a significant decrease in the influx rate (Fig. 6B–D). Notably, these alterations in spontaneous Ca^2+^ oscillations were partially recovered in cells treated with the 1P protocol. To further strengthen our findings, siRNA experiments were carried out in two additional hiPSCs cell lines that also showed MAO-A upregulation during mesoderm-cardiac specification and cardiomyocyte formation (Suppl. Figure 1B). Also in this case, the reduction in AKT/GSK3β phosphorylation levels and sarcomere disarray were causally related to MAO-A silencing (Suppl. Figure 4D and Suppl. Figure 5A–B). Moreover, in cells treated with MAO-A siRNA (2P) we observed a significant increase in the frequency of spontaneous oscillations in the cytosolic Ca^2+^ and a significant decrease in the peak amplitude (Suppl. Figure 5C–D). The 2P protocol induced an impairment in response to caffeine in KD cells, with a significant decrease in the Ca^2+^ influx rate (Suppl. Figure 5C–E). Altogether, these results strongly support MAO-dependent ROS generation as a regulator of AKT and WNT pathways during cardiac differentiation. In particular, the increase in MAO-A-dependent ROS formation during the transition from cardio-mesoderm to cardiomyocytes is required for the correct differentiation of hiPSCs into cardiac cells.

## Discussion

This study identifies MAO-A dependent ROS formation as an important process contributing to cardiomyocyte lineage commitment. MAO-A KO/KD hiPSC-CMs exhibit impaired sarcomere structure and function, along with lower mitochondrial ROS levels. Lack of MAO-A dependent ROS formation leads to a decrease in AKT/GSK3β phosphorylation, *WNT* expression and downstream activation of cardiac transcription factors *MESP1* and *NKX2.5*. This causal relationship was further supported by showing that either exogenous H_2_O_2_ administration to MAO-A KO cells or MAO-A re-expression improved AKT/GSK3β phosphorylation, *NKX2.5* and *WNT3A* transcript abundance, and rescued structural disarray and alterations in Ca^2+^ homeostasis.

ROS are key players in cardiomyogenesis and differentiation of stem cells into cardiomyocytes in vitro [7, 9, 21, 40]. In addition, mitochondrial ROS formation has been linked to the activation of the *NOX4* gene and p38 phosphorylation [12]. Indeed, mitochondria-targeted antioxidants blocked cardiac differentiation [12], suggesting that alterations in the mitochondrial redox equilibrium during cardiac specification compromise this process. Here we identify the mitochondrial flavoenzyme MAO-A as a prominent source of mitochondrial ROS that play an important role in cardiomyocyte lineage commitment. Genetic or pharmacological inhibition of MAO-A did not block the formation of contracting cardiomyocytes, unlike ROS scavengers, pointing to the possibility that a cross-talk between MAO-A and other ROS sources might exist. Nevertheless, myofibrillogenesis was impaired in MAO-A mutant cardiomyocytes, showing sarcomere structure disarray, reduction of the myofibrillar protein MYHC6, as well as aberrant intracellular Ca^2+^ cycling. While cytosolic Ca^2+^ transients were dramatically reduced in MAO-A KO hiPSC-CMs, no differences were observed in the mitochondrial Ca^2+^ levels. In addition, gene expression of Ca^2+^ handling proteins, the phosphorylation status of PLN and the distance/interaction between mitochondria and SR all remained unaffected by MAO-A deletion. This suggests that mitochondrial Ca^2+^ uptake does not impact on cytosolic Ca^2+^ levels, at least in this model. Moreover, we unequivocally demonstrated that MAOA-dependent ROS formation is important between days 4 and 6 of cardiomyocyte differentiation, i.e., during cardiac specification. These findings are in line with previous studies suggesting that redox signaling during cardiomyogenesis might be both stage- and dose-dependent and that the commitment of cardiac progenitor cells toward the cardiac lineage in the early embryo is promoted by ROS [21, 26]. Our experiments suggest that the potential for MAO-dependent ROS formation greatly depends on the substrate availability. Further studies will elucidate whether baseline MAO-A activity is sufficient to drive cardiomyocyte differentiation or whether induction of MAO-A expression is the key element regulating this process.

Reduced autophagy, alteration in GATA4 activity and myofibrillar protein expression are frequently signs of a maladaptive response to the underlying cardiac dysfunction in several cardiac pathologies [13, 19, 52, 72]. Such alterations were also observed in MAO-A KO hiPSC-CMs and are likely the result of an adaptive response to an impairment in differentiation process. A compensatory increase in GATA4 activity is required for the maintenance of cardiac function in the postnatal heart or following stress stimuli [5, 53]. Indeed, here we observed GATA4 upregulation in MAO-A KO hiPSC-CMs as compared to control cells. These findings are in line with another study showing that cardiomyogenesis was inhibited and GATA4 levels increased following administration of ROS scavengers to embryonic stem cells (ESCs) during cardiac differentiation [62].

*NKX2.5* is directly involved in the regulation of several processes that collectively contribute to cardiomyogenesis and morphogenesis of the mature heart [22]. Moreover, expression of *MESP1*, a master regulator of multipotent cardiovascular progenitor specification [6], is promoted by GSK3β activation [66]. In our experimental setting, AKT and GSK3β phosphorylation were reduced in MAO-A KO or KD cells starting from day 4. AKT phosphorylation levels change dynamically during cardiomyogenesis, suggesting that AKT inhibition occurs during mesoderm specification (day 2) and partial AKT activation is required for cardiac lineage commitment (from day 4 on). Our results suggest that ROS/MAO-A dependent AKT activation plays a pivotal role during cardiac mesoderm formation (day 4). Given that MAO-A dependent ROS formation is down-regulated throughout differentiation, present results imply that AKT activation starts relying on ROS produced by MAO-A during cardiac precursors formation. This decreased activation of AKT/GSK3β signaling in the absence of MAO-A resulted in lower levels of both *MESP1* and *NKX2.5*, suggesting an altered cardiac commitment. Furthermore, we observed a remarkable down regulation in *WNT3A* expression levels at day 6 in KO cells, in parallel with a significant reduction of both *WNT3* and *WNT11*.

Endogenous canonical *WNT* (*WNT3* and *3A*) levels during cell fate determination are causally related to *MESP1* expression, mesodermal commitment and patterning toward cardiac mesoderm [41, 46]. On the other hand, up-regulation of non-canonical *WNT* expression (*WNT11*) in the later phase of differentiation plays an important role in cardiac development [69]. Notably, stimulation of canonical WNT pathway between days 4–6 with the soluble ligand WNT3A augments cardiac differentiation from ESCs [37]. Accordingly, inhibition of WNT pathway with dickkopf-related protein 1 during this window drastically compromises cardiomyocyte differentiation [56], suggesting that timely canonical WNT signaling is required for cardiomyocyte formation. Moreover, cardiac progenitors are formed at this stage, and the following maturation into cardiomyocytes is prompted by specific cardiac regulators. In human fetal cardiovascular progenitor cells derived from ESC lines, the self-renewal capacity and expansion of the cells is promoted and sustained by WNT3A [8]. In our model, pharmacological AKT inhibition with MK-2206 did not alter expression levels of *WNT3* and *WNT3A*, indicating that downregulation of the canonical *WNT* genes in MAO-A KO cells was not under the direct control of AKT. However, when we re-expressed MAO-A protein or when MAO-A KO cells have been exposed to a pulse of H_2_O_2_, levels of *WNT* genes increased significantly. In addition, specific mitochondrial ROS scavenging by mitoTEMPO significantly reduced *WNT3A* expression. These results suggest that persistent alteration of the mitochondrial redox balance in MAO-A KO cells leads to a parallel decrease in AKT and WNT signaling.

To date, the physiological involvement of MAOs in differentiation processes and development has been described in the brain, where MAO-A inhibition severely affects neurogenesis [75]. This effect was explained by an elevation in serotonin levels since monoaminergic transmitter system is important for brain development, but the possible involvement of ROS was not assessed. The present study provides evidence showing that MAO-A related ROS generation is necessary for proper activation of the AKT and WNT signaling pathways in the early stages of human cardiomyogenesis and for the differentiation of fully functional cardiomyocytes. Despite the extensively demonstrated efficacy of MAO inhibition in preventing cardiac damage in several pathological conditions [15], present findings indicate a dual, hormetic role for ROS produced by MAO-A.

### Supplementary Information

Below is the link to the electronic supplementary material.

Supplementary file1 (DOCX 5006 KB)

## Data Availability

The analyzed datasets are available from the corresponding author upon reasonable request.

## Abbreviations

1P/2P: 1 Pulse/2 pulses
AKT: AKT serine/threonine kinase
CMs: Cardiomyocytes
ERMICC: ER–mitochondria contact coefficient
ER/SR: Endoplasmic/sarcoplasmic reticulum
ESCs: Embryonic stem cells
FCCP: Carbonyl cyanide 4-(trifluoromethoxy)phenylhydrazone
GATA4: GATA binding protein 4
GSK3β: Glycogen synthase kinase 3ß
hiPSCs: Human induced pluripotent stem cells
KO/KD: Knock out/knock down
LC3B: Microtubule-associated protein 1 light chain 3ß
MAO-A/B: Monoamine oxidase A/B
MCU: Mitochondrial calcium uniporter
MESP1: Mesoderm posterior BHLH transcription factor 1
MM: Maturation medium
MMTV: Integration site family protein
MPG: N-(2-mercaptopropionyl)-glycine
MTR: Mitotracker Red CM-H_2_XRos
MYHC: Myosin heavy chain
NCX1: Na^+^/Ca^2+^-exchange protein 1
NKX2.5: NK2 homeobox 5
p38: Mitogen-activated protein kinase
p62: Sequestosome 1
PLN: Phospholamban
qRT-PCR: Quantitative real time PCR
RCAN1: Regulator of calcineurin 1
ROS: Reactive oxygen species
RYR2: Ryanodine receptor 2
SERCA2A: Sarco-endoplasmic reticulum calcium ATPase 2A
TEM: Transmission electron microscopy
TMRM: Tetramethylrhodamine
WNT: Wingless-related integration site
Wnt-C59: Porcupine (PORCN) inhibitor

## References

[1] Antonucci S, Di Sante M, Tonolo F, Pontarollo L, Scalcon V, Alanova P, Menabo R, Carpi A, Bindoli A, Rigobello MP, Giorgio M, Kaludercic N, Di Lisa F (2021). The determining role of mitochondrial reactive oxygen species generation and monoamine oxidase activity in doxorubicin-induced cardiotoxicity. Antioxid Redox Signal.

[2] Berniakovich I, Trinei M, Stendardo M, Migliaccio E, Minucci S, Bernardi P, Pelicci PG, Giorgio M (2008). p66Shc-generated oxidative signal promotes fat accumulation. J Biol Chem.

[3] Bigarella CL, Liang R, Ghaffari S (2014). Stem cells and the impact of ROS signaling. Development.

[4] Binda C, Newton-Vinson P, Hubalek F, Edmondson DE, Mattevi A (2002). Structure of human monoamine oxidase B, a drug target for the treatment of neurological disorders. Nat Struct Biol.

[5] Bisping E, Ikeda S, Kong SW, Tarnavski O, Bodyak N, McMullen JR, Rajagopal S, Son JK, Ma Q, Springer Z, Kang PM, Izumo S, Pu WT (2006). Gata4 is required for maintenance of postnatal cardiac function and protection from pressure overload-induced heart failure. Proc Natl Acad Sci U S A.

[6] Bondue A, Lapouge G, Paulissen C, Semeraro C, Iacovino M, Kyba M, Blanpain C (2008). Mesp1 acts as a master regulator of multipotent cardiovascular progenitor specification. Cell Stem Cell.

[7] Boopathy AV, Pendergrass KD, Che PL, Yoon YS, Davis ME (2013). Oxidative stress-induced Notch1 signaling promotes cardiogenic gene expression in mesenchymal stem cells. Stem Cell Res Ther.

[8] Bu L, Jiang X, Martin-Puig S, Caron L, Zhu S, Shao Y, Roberts DJ, Huang PL, Domian IJ, Chien KR (2009). Human ISL1 heart progenitors generate diverse multipotent cardiovascular cell lineages. Nature.

[9] Buggisch M, Ateghang B, Ruhe C, Strobel C, Lange S, Wartenberg M, Sauer H (2007). Stimulation of ES-cell-derived cardiomyogenesis and neonatal cardiac cell proliferation by reactive oxygen species and NADPH oxidase. J Cell Sci.

[10] Cagnin S, Brugnaro M, Millino C, Pacchioni B, Troiano C, Di Sante M, Kaludercic N (2022). Monoamine oxidase-dependent pro-survival signaling in diabetic hearts is mediated by miRNAs. Cells.

[11] Chandel NS (2015). Evolution of mitochondria as signaling organelles. Cell Metab.

[12] Crespo FL, Sobrado VR, Gomez L, Cervera AM, McCreath KJ (2010). Mitochondrial reactive oxygen species mediate cardiomyocyte formation from embryonic stem cells in high glucose. Stem Cells.

[13] Dennemarker J, Lohmuller T, Muller S, Aguilar SV, Tobin DJ, Peters C, Reinheckel T (2010). Impaired turnover of autophagolysosomes in cathepsin L deficiency. Biol Chem.

[14] Deshwal S, Antonucci S, Kaludercic N, Di Lisa F (2018). Measurement of mitochondrial ROS formation. Methods Mol Biol.

[15] Deshwal S, Di Sante M, Di Lisa F, Kaludercic N (2017). Emerging role of monoamine oxidase as a therapeutic target for cardiovascular disease. Curr Opin Pharmacol.

[16] Deshwal S, Forkink M, Hu CH, Buonincontri G, Antonucci S, Di Sante M, Murphy MP, Paolocci N, Mochly-Rosen D, Krieg T, Di Lisa F, Kaludercic N (2018). Monoamine oxidase-dependent endoplasmic reticulum-mitochondria dysfunction and mast cell degranulation lead to adverse cardiac remodeling in diabetes. Cell Death Differ.

[17] Di Lisa F, Kaludercic N, Carpi A, Menabo R, Giorgio M (2009). Mitochondrial pathways for ROS formation and myocardial injury: the relevance of p66(Shc) and monoamine oxidase. Basic Res Cardiol.

[18] Dickson BJ, Gatie MI, Spice DM, Kelly GM (2017). NOX1 and NOX4 are required for the differentiation of mouse F9 cells into extraembryonic endoderm. PLoS One.

[19] Dirkx E, da Costa Martins PA, De Windt LJ (2013). Regulation of fetal gene expression in heart failure. Biochim Biophys Acta.

[20] Drawnel FM, Boccardo S, Prummer M, Delobel F, Graff A, Weber M, Gerard R, Badi L, Kam-Thong T, Bu L, Jiang X, Hoflack JC, Kiialainen A, Jeworutzki E, Aoyama N, Carlson C, Burcin M, Gromo G, Boehringer M, Stahlberg H, Hall BJ, Magnone MC, Kolaja K, Chien KR, Bailly J, Iacone R (2014). Disease modeling and phenotypic drug screening for diabetic cardiomyopathy using human induced pluripotent stem cells. Cell Rep.

[21] Drenckhahn JD (2011). Heart development: mitochondria in command of cardiomyocyte differentiation. Dev Cell.

[22] Flaherty MP, Kamerzell TJ, Dawn B (2012). Wnt signaling and cardiac differentiation. Prog Mol Biol Transl Sci.

[23] Forman HJ, Fukuto JM, Torres M (2004). Redox signaling: thiol chemistry defines which reactive oxygen and nitrogen species can act as second messengers. Am J Physiol Cell Physiol.

[24] Funato Y, Michiue T, Asashima M, Miki H (2006). The thioredoxin-related redox-regulating protein nucleoredoxin inhibits Wnt-beta-catenin signalling through dishevelled. Nat Cell Biol.

[25] Hauptmann N, Grimsby J, Shih JC, Cadenas E (1996). The metabolism of tyramine by monoamine oxidase A/B causes oxidative damage to mitochondrial DNA. Arch Biochem Biophys.

[26] Hom JR, Quintanilla RA, Hoffman DL, de Mesy Bentley KL, Molkentin JD, Sheu SS, Porter GA (2011). The permeability transition pore controls cardiac mitochondrial maturation and myocyte differentiation. Dev Cell.

[27] Hsu PD, Scott DA, Weinstein JA, Ran FA, Konermann S, Agarwala V, Li Y, Fine EJ, Wu X, Shalem O, Cradick TJ, Marraffini LA, Bao G, Zhang F (2013). DNA targeting specificity of RNA-guided Cas9 nucleases. Nat Biotechnol.

[28] Ingwall JS (2009). Energy metabolism in heart failure and remodelling. Cardiovasc Res.

[29] Kajla S, Mondol AS, Nagasawa A, Zhang Y, Kato M, Matsuno K, Yabe-Nishimura C, Kamata T (2012). A crucial role for Nox 1 in redox-dependent regulation of Wnt-beta-catenin signaling. FASEB J.

[30] Kaludercic N, Carpi A, Nagayama T, Sivakumaran V, Zhu G, Lai EW, Bedja D, De Mario A, Chen K, Gabrielson KL, Lindsey ML, Pacak K, Takimoto E, Shih JC, Kass DA, Di Lisa F, Paolocci N (2014). Monoamine oxidase B prompts mitochondrial and cardiac dysfunction in pressure overloaded hearts. Antioxid Redox Signal.

[31] Kaludercic N, Maiuri MC, Kaushik S, Fernandez AF, de Bruijn J, Castoldi F, Chen Y, Ito J, Mukai R, Murakawa T, Nah J, Pietrocola F, Saito T, Sebti S, Semenzato M, Tsansizi L, Sciarretta S, Madrigal-Matute J (2020). Comprehensive autophagy evaluation in cardiac disease models. Cardiovasc Res.

[32] Kaludercic N, Mialet-Perez J, Paolocci N, Parini A, Di Lisa F (2014). Monoamine oxidases as sources of oxidants in the heart. J Mol Cell Cardiol.

[33] Kaludercic N, Takimoto E, Nagayama T, Feng N, Lai EW, Bedja D, Chen K, Gabrielson KL, Blakely RD, Shih JC, Pacak K, Kass DA, Di Lisa F, Paolocci N (2010). Monoamine oxidase A-mediated enhanced catabolism of norepinephrine contributes to adverse remodeling and pump failure in hearts with pressure overload. Circ Res.

[34] Kanamori H, Takemura G, Goto K, Maruyama R, Tsujimoto A, Ogino A, Takeyama T, Kawaguchi T, Watanabe T, Fujiwara T, Fujiwara H, Seishima M, Minatoguchi S (2011). The role of autophagy emerging in postinfarction cardiac remodelling. Cardiovasc Res.

[35] Kempf H, Lecina M, Ting S, Zweigerdt R, Oh S (2011). Distinct regulation of mitogen-activated protein kinase activities is coupled with enhanced cardiac differentiation of human embryonic stem cells. Stem Cell Res.

[36] Krenz M, Robbins J (2004). Impact of beta-myosin heavy chain expression on cardiac function during stress. J Am Coll Cardiol.

[37] Kwon C, Arnold J, Hsiao EC, Taketo MM, Conklin BR, Srivastava D (2007). Canonical Wnt signaling is a positive regulator of mammalian cardiac progenitors. Proc Natl Acad Sci U S A.

[38] Kwon SK, Sando R, Lewis TL, Hirabayashi Y, Maximov A, Polleux F (2016). LKB1 regulates mitochondria-dependent presynaptic calcium clearance and neurotransmitter release properties at excitatory synapses along cortical axons. PLoS Biol.

[39] Lee S, Tak E, Lee J, Rashid MA, Murphy MP, Ha J, Kim SS (2011). Mitochondrial H2O2 generated from electron transport chain complex I stimulates muscle differentiation. Cell Res.

[40] Li J, Stouffs M, Serrander L, Banfi B, Bettiol E, Charnay Y, Steger K, Krause KH, Jaconi ME (2006). The NADPH oxidase NOX4 drives cardiac differentiation: Role in regulating cardiac transcription factors and MAP kinase activation. Mol Biol Cell.

[41] Lian X, Hsiao C, Wilson G, Zhu K, Hazeltine LB, Azarin SM, Raval KK, Zhang J, Kamp TJ, Palecek SP (2012). Robust cardiomyocyte differentiation from human pluripotent stem cells via temporal modulation of canonical Wnt signaling. Proc Natl Acad Sci U S A.

[42] Lin L, Cui L, Zhou W, Dufort D, Zhang X, Cai CL, Bu L, Yang L, Martin J, Kemler R, Rosenfeld MG, Chen J, Evans SM (2007). Beta-catenin directly regulates Islet1 expression in cardiovascular progenitors and is required for multiple aspects of cardiogenesis. Proc Natl Acad Sci U S A.

[43] Machackova J, Barta J, Dhalla NS (2006). Myofibrillar remodeling in cardiac hypertrophy, heart failure and cardiomyopathies. Can J Cardiol.

[44] Malandraki-Miller S, Lopez CA, Al-Siddiqi H, Carr CA (2018). Changing metabolism in differentiating cardiac progenitor cells-can stem cells become metabolically flexible cardiomyocytes?. Front Cardiovasc Med.

[45] Maryanovich M, Gross A (2013). A ROS rheostat for cell fate regulation. Trends Cell Biol.

[46] Mehta A, Ramachandra CJ, Sequiera GL, Sudibyo Y, Nandihalli M, Yong PJ, Koh CH, Shim W (2014). Phasic modulation of Wnt signaling enhances cardiac differentiation in human pluripotent stem cells by recapitulating developmental ontogeny. Biochim Biophys Acta.

[47] Mialet-Perez J, Bianchi P, Kunduzova O, Parini A (2007). New insights on receptor-dependent and monoamine oxidase-dependent effects of serotonin in the heart. J Neural Transm (Vienna).

[48] Miyata S, Minobe W, Bristow MR, Leinwand LA (2000). Myosin heavy chain isoform expression in the failing and nonfailing human heart. Circ Res.

[49] Murphy MP (2009). How mitochondria produce reactive oxygen species. Biochem J.

[50] Murphy MP (2012). Modulating mitochondrial intracellular location as a redox signal. Sci Signal.

[51] Naito AT, Akazawa H, Takano H, Minamino T, Nagai T, Aburatani H, Komuro I (2005). Phosphatidylinositol 3-kinase-Akt pathway plays a critical role in early cardiomyogenesis by regulating canonical Wnt signaling. Circ Res.

[52] Nakai A, Yamaguchi O, Takeda T, Higuchi Y, Hikoso S, Taniike M, Omiya S, Mizote I, Matsumura Y, Asahi M, Nishida K, Hori M, Mizushima N, Otsu K (2007). The role of autophagy in cardiomyocytes in the basal state and in response to hemodynamic stress. Nat Med.

[53] Oka T, Maillet M, Watt AJ, Schwartz RJ, Aronow BJ, Duncan SA, Molkentin JD (2006). Cardiac-specific deletion of Gata4 reveals its requirement for hypertrophy, compensation, and myocyte viability. Circ Res.

[54] Owusu-Ansah E, Banerjee U (2009). Reactive oxygen species prime Drosophila haematopoietic progenitors for differentiation. Nature.

[55] Parikh A, Wu J, Blanton RM, Tzanakakis ES (2015). Signaling pathways and gene regulatory networks in cardiomyocyte differentiation. Tissue Eng Part B Rev.

[56] Peng G, Han JJ (2018) Regulatory network characterization in development: challenges and opportunities. F1000Res7 10.12688/f1000research.15271.1 10.12688/f1000research.15271.1 PMC6144950 30271577

[57] Rharass T, Lemcke H, Lantow M, Kuznetsov SA, Weiss DG, Panakova D (2014). Ca2+-mediated mitochondrial reactive oxygen species metabolism augments Wnt/beta-catenin pathway activation to facilitate cell differentiation. J Biol Chem.

[58] Romero A, Marco-Contelles J, Ramos E (2020). Highlights of ASS234: a novel and promising therapeutic agent for Alzheimer's disease therapy. Neural Regen Res.

[59] Sanjana NE, Shalem O, Zhang F (2014). Improved vectors and genome-wide libraries for CRISPR screening. Nat Methods.

[60] Saura J, Kettler R, Da Prada M, Richards JG (1992). Quantitative enzyme radioautography with 3H-Ro 41–1049 and 3H-Ro 19–6327 in vitro: localization and abundance of MAO-A and MAO-B in rat CNS, peripheral organs, and human brain. J Neurosci.

[61] Schindelin J, Arganda-Carreras I, Frise E, Kaynig V, Longair M, Pietzsch T, Preibisch S, Rueden C, Saalfeld S, Schmid B, Tinevez JY, White DJ, Hartenstein V, Eliceiri K, Tomancak P, Cardona A (2012). Fiji: an open-source platform for biological-image analysis. Nat Methods.

[62] Schmelter M, Ateghang B, Helmig S, Wartenberg M, Sauer H (2006). Embryonic stem cells utilize reactive oxygen species as transducers of mechanical strain-induced cardiovascular differentiation. FASEB J.

[63] Shalem O, Sanjana NE, Hartenian E, Shi X, Scott DA, Mikkelson T, Heckl D, Ebert BL, Root DE, Doench JG, Zhang F (2014). Genome-scale CRISPR-Cas9 knockout screening in human cells. Science.

[64] Shearer RF, Saunders DN (2015). Experimental design for stable genetic manipulation in mammalian cell lines: lentivirus and alternatives. Genes Cells.

[65] Sheehy SP, Huang S, Parker KK (2009). Time-warped comparison of gene expression in adaptive and maladaptive cardiac hypertrophy. Circ Cardiovasc Genet.

[66] Si X, Chen W, Guo X, Chen L, Wang G, Xu Y, Kang J (2013). Activation of GSK3beta by Sirt2 is required for early lineage commitment of mouse embryonic stem cell. PLoS One.

[67] Sivasubramaniam SD, Finch CC, Rodriguez MJ, Mahy N, Billett EE (2003). A comparative study of the expression of monoamine oxidase-A and -B mRNA and protein in non-CNS human tissues. Cell Tissue Res.

[68] Son SY, Ma J, Kondou Y, Yoshimura M, Yamashita E, Tsukihara T (2008). Structure of human monoamine oxidase A at 2.2-A resolution: the control of opening the entry for substrates/inhibitors. Proc Natl Acad Sci U S A.

[69] Terami H, Hidaka K, Katsumata T, Iio A, Morisaki T (2004). Wnt11 facilitates embryonic stem cell differentiation to Nkx2.5-positive cardiomyocytes. Biochem Biophys Res Commun.

[70] Tohyama S, Hattori F, Sano M, Hishiki T, Nagahata Y, Matsuura T, Hashimoto H, Suzuki T, Yamashita H, Satoh Y, Egashira T, Seki T, Muraoka N, Yamakawa H, Ohgino Y, Tanaka T, Yoichi M, Yuasa S, Murata M, Suematsu M, Fukuda K (2013). Distinct metabolic flow enables large-scale purification of mouse and human pluripotent stem cell-derived cardiomyocytes. Cell Stem Cell.

[71] Tormos KV, Anso E, Hamanaka RB, Eisenbart J, Joseph J, Kalyanaraman B, Chandel NS (2011). Mitochondrial complex III ROS regulate adipocyte differentiation. Cell Metab.

[72] van Berlo JH, Elrod JW, Aronow BJ, Pu WT, Molkentin JD (2011). Serine 105 phosphorylation of transcription factor GATA4 is necessary for stress-induced cardiac hypertrophy in vivo. Proc Natl Acad Sci U S A.

[73] Vikram A, Kim YR, Kumar S, Naqvi A, Hoffman TA, Kumar A, Miller FJ, Kim CS, Irani K (2014). Canonical Wnt signaling induces vascular endothelial dysfunction via p66Shc-regulated reactive oxygen species. Arterioscler Thromb Vasc Biol.

[74] Villeneuve C, Guilbeau-Frugier C, Sicard P, Lairez O, Ordener C, Duparc T, De Paulis D, Couderc B, Spreux-Varoquaux O, Tortosa F, Garnier A, Knauf C, Valet P, Borchi E, Nediani C, Gharib A, Ovize M, Delisle MB, Parini A, Mialet-Perez J (2013). p53-PGC-1alpha pathway mediates oxidative mitochondrial damage and cardiomyocyte necrosis induced by monoamine oxidase-A upregulation: role in chronic left ventricular dysfunction in mice. Antioxid Redox Signal.

[75] Wang CC, Borchert A, Ugun-Klusek A, Tang LY, Lui WT, Chu CY, Billett E, Kuhn H, Ufer C (2011). Monoamine oxidase a expression is vital for embryonic brain development by modulating developmental apoptosis. J Biol Chem.

[76] Wei H, Cong X (2018). The effect of reactive oxygen species on cardiomyocyte differentiation of pluripotent stem cells. Free Radic Res.

[77] Zhan LF, Huang YQ, Chen Q, Ma XD (2017). Effect of Monoamine Oxidase Inhibitor Phenelzine on Proliferation of Mantle Cell Lymphoma and Its Mechanism. Zhongguo Shi Yan Xue Ye Xue Za Zhi.

[78] Zhao M, Tang Y, Zhou Y, Zhang J (2019). Deciphering Role of Wnt Signalling in Cardiac Mesoderm and Cardiomyocyte Differentiation from Human iPSCs: Four-dimensional control of Wnt pathway for hiPSC-CMs differentiation. Sci Rep.

[79] Zhao Q, Sun Q, Zhou L, Liu K, Jiao K (2019). Complex Regulation of Mitochondrial Function During Cardiac Development. J Am Heart Assoc.

