# Supplementary material for: Monoamine oxidase A-dependent ROS formation modulates human cardiomyocyte differentiation through AKT and WNT activation
Source: Basic Res Cardiol. 2023 Jan 20;118(1):4. doi: 10.1007/s00395-023-00977-4 (PMC9859871; doi:10.1007/s00395-023-00977-4)
Supplement: Supplementary file 1 — Supplementary file1 (DOCX 5006 KB) [file 395_2023_977_MOESM1_ESM.docx]

**Supplementary Material**

**Expanded Materials and Methods**

**hiPSCs maintenance, cardiac differentiation and maturation**

SCVI15 hiPSCs line was kindly provided by Professor [Joseph C. Wu,](https://profiles.stanford.edu/joseph-wu) (Stanford Cardiovascular Institute, USA). ATCC-BYS0112 hiPSCs were purchased from ATCC (ATCC, ACS-1026). UST000013 hiPSCs line was purchased from uSTEM s.r.l. Cells were maintained in Essential 8 Flex Medium (Thermo Fisher Scientific) on 1:100 Geltrex LDEV-Free Reduced Growth Factor Basement Membrane Matrix (Thermo Fisher Scientific) and passaged every 4 days using 0.5 mM ethylenediaminetetraacetic acid (EDTA) (Thermo Fisher Scientific) in Dulbecco’s PBS (DPBS) without Ca^2+^ and Mg^2+^ (Life Technologies) for 5 min at 37°C in the presence of 5% CO_2_. Cells were expanded in 6-well plates by passaging 1:8. hiPSCs were differentiated in cardiomyocytes (CMs) by means of the STEMdiff^TM^ Cardiomyocyte Differentiation Kit (Stemcell Technologies) following manufacturer’s instructions. Briefly, hiPSCs were plated at the density of 3.5 x 10^5^ cells/well in a 12-well plate or at the density of 3 x 10^4^ cells/well in 96-well plate. After reaching 90% confluence, cells were incubated with the induction medium A for the mesodermal commitment. Two days after, cardiac mesoderm commitment was achieved by replacing medium A with medium B. After four days, the medium was replaced with medium C for the cardiac induction. After eight days from the beginning of the differentiation protocol, the induction medium C was replaced with maintenance medium and cells were re-fed every 48h. Cells differentiated with this protocol display spontaneous beating usually on day 8 and robust contracting syncytium by day 10. On day 14, beating cardiomyocytes have been selected as described before [[8](#_ENREF_8)]. Briefly, cells were cultured in glucose-free DMEM (no glucose, no pyruvate, Life Technologies) supplemented with 4 mM Na-DL-Lactate (Sigma) for 48h. Medium was renewed every day and it was replaced after 2 days with maintenance medium. Cardiomyocytes were used for the experiments after 4 days from the selection protocol [[8](#_ENREF_8)] unless otherwise specified in the text. Cells were exposed to maturation medium (MM) for 3 days. MM is Dulbecco’s modified Eagle’s medium no glucose (GIBCO), 10 mM HEPES, 2 mM L-carnitine, 5 mM creatine, 5 mM taurine, 1 mM non-essential amino acids (GIBCO), 1x insulin-transferrin-selenium (Sigma-Aldrich), and linoleic-oleic acid (Sigma-Aldrich) [[1](#_ENREF_1)].

**Design and construction of CRISPRs targeting human MAO-A**

Lentiviral CRISPR/Cas9 expression constructs containing the guides for human MAO-A were generated using the lentiCRISPRv2 one vector system [[4](#_ENREF_4), [6](#_ENREF_6)]. Briefly, oligos were synthetized after identification of suitable target sites using Cas9 target design tool (ThermoFisher Scientific). Three different oligos containing the 20-bp MAO-A target sequence with BsmBI-compatible sticky ends were annealed to the complementary oligos, and cloned into BsmBI-digested lentiCRISPRv2 plasmid (Addgene). The following oligos have been tested:

1. sgRNA-1/2
   1. hMAO-A oligo1: CACCGCAAGAGAAGGCGAGTATCGC
   2. hMAO-A oligo2: AAACGCGATACTCGCCTTCTCTTGC
2. sgRNA-3/4
   1. hMAO-A oligo3: CACCGATGTTCGACGTAGTCGTGAT
   2. hMAO-A oligo4: AAACATCACGACTACGTCGAACATC
3. sgRNA-5/6
   1. hMAO-A oligo5: CACCGGGCTCAACATGCTGACAAAT
   2. hMAO-A oligo6: AAACATTTGTCAGCATGTTGAGCCC

sgRNA-3/4 was chosen as the most effective one and used to generate stable isogenic MAO-A KO hiPSCs line.

**Lentiviral production and hiPSCs infection**

To produce lentiviral particles, HEK293T cells were plated on 10 cm petri dishes at a density of 2.5 x 10^6^ cells/dish. Cells were transfected using Lipofectamine 3000 reagent (Thermo Fisher Scientific) at 70–80% confluency following manufacturer's recommended protocol. For each transfection, four different plasmids were used to generate lentiviral particles (i.e., 5 µg LentiCRISPRv2_hMAO-A plasmid, 1.5 µg envelope plasmid pMD2.G, 2.5 µg pMDLg/pRRE plasmid and 1.25 µg packaging vector pRSV-Rev plasmid) [[7](#_ENREF_7)]. To determine the efficacy of gene knockout by lentiCRISPR transduction, 3 different single guide RNAs (sgRNA-1/2, sgRNA-3/4, sgRNA-5/6) targeting MAO-A locus were tested in HEK293T cells. A non-MAO-A-targeting control sgRNAs against enhanced green fluorescent protein (EGFP) were used as control (Addgene 51760) [[6](#_ENREF_6)]. MAO-A and MAO-B expression levels were evaluated by western blot analysis after puromycin selection. Wells showing undetectable levels of MAO-A mRNA/protein were chosen for knock-out cell line expansion and experiments.

**Live cell imaging**

hiPSC-CMs were differentiated in black µ-Plate 96-well plates (Ibidi), and experiments were carried out in an extracellular medium (containing: 137 mM NaCl, 5 mM KCl, 0.4 mM MgCl_2_, 0.5 mM MgSO_4_, 0.4 mM KH_2_PO_4_, 0.4 mM Na_2_HPO_4_, 5.5 mM glucose, and 20 mM MOPS) at pH 7.4 (adjusted with NaOH) and at 37°C.

Images were acquired using an inverted fluorescence microscope (Leica DMI6000B equipped with a DFC365FX camera) with PL APO 40x/1.25 oil objective. Fluorescence intensity was quantified using the Fiji distribution of the Java-based image processing program ImageJ [[5](#_ENREF_5)], and background signal was subtracted from all analysed regions of interest. For Ca^2+^ imaging, traces were analysed using the “Peak Analyzer” tool of Origin Pro 9.1.

To monitor mitochondrial ROS, cells were incubated with 25 nM MitoTracker Red CM-H_2_XRos (MTR, Thermo Fisher Scientific) for 30 min at 37°C, in the presence of 5% CO_2_. Images were collected at day 0 (hiPSCs), day 2 (mesodermal commitment), day 4 (cardiac commitment), and day 20 (cardiomyocytes). Since the accumulation of MTR in cells can vary between different experimental days, data were normalized to the basal value determined in WT hiPSCs.

To monitor cytosolic Ca^2+^ transients, cells were loaded with Fluo-4 acetoxymethyl ester (Thermo Fisher Scientific), 0.01% w/v pluronic F-127 (Sigma), and 250 µM sulfinpyrazone (Sigma) for 40 min at 37°C in a humidified incubator followed by 20 min of de-esterification. Images have been acquired every 200 ms. A typical experiment consists in the recordings of the spontaneous beating for 1 minute. At least three different fields of view have been acquired for each sample. To evaluate the release of Ca^2+^ from the SR, a pulse of 10 mM caffeine was added to the cells. Since the accumulation of Fluo-4 in NRVMs can vary between different preparations, data were expressed as ΔF/F0 and then normalized to hiPSC WT.

To monitor mitochondrial membrane potential (ΔΨm), cells were incubated with 25 nM tetramethylrhodamine (TMRM, Thermo Fisher Scientific) in the presence of 1.6 µM cyclosporin H for 30 min at 37°C in a humidified incubator. Images were acquired before and after the addition of 4 µM carbonyl cyanide-p-trifluoromethoxyphenylhydrazone (FCCP, Sigma) [[2](#_ENREF_2)]. Images were collected at day 0 (hiPSCs), day 2 (mesodermal commitment), day 4 (cardiac commitment), and day 20 (cardiomyocytes). Fluorescence values were expressed as ΔF (F_0_/F_FCCP_), and results were normalized to hiPSC WT basal value.

**Immunocytochemistry**

hiPSCs and hiPSC-CMs were prepared for staining using Cardiomyocyte Immunocytochemistry Kit (Thermo Fisher Scientific) following manufacturer’s instruction. Sarcomeres were stained using anti-α-sarcomeric actinin antibody (Sigma; 1:500, mouse) over-night (O/N) at 4°C. The day after, samples were incubated for 1 h at room temperature with Alexa Fluor 488 conjugated anti-mouse (Life Technologies, 1:250) and phalloidin TRITC conjugated (Sigma Aldrich, 1:500) for actin staining. Coverslips were mounted using NucBlueTM Fixed Cell ReadyProbesTM Reagent with DAPI to stain nuclei (Invitrogen). Images were collected using Zeiss LSM 700 confocal system equipped with a PlanApo 40x/1.2 oil objective at 2048x2048 pixels per image with a 100 Hz acquisition rate. Images were analysed using the Fiji distribution of the Java-based image processing program ImageJ [[5](#_ENREF_5)].

**Western blot analysis**

hiPSCs and hiPSC-CMs were homogenized in RIPA lysis buffer (EMD Millipore) containing protease and phosphatase inhibitors (Roche). Protein concentration was determined using BCA protein assay (Pierce). Proteins were separated using SDS–PAGE (Invitrogen) and transferred to nitrocellulose membrane (Bio-Rad). Following incubation with primary and secondary HRP-conjugated antibody (Bio-Rad), bands were detected by the Pierce^TM^ ECL Western Blotting Substrate using the UVITEC Cambridge mini HD instrument. Densitometry analysis was performed using the Fiji distribution of the Java-based image processing program ImageJ [[5](#_ENREF_5)]. The following antibodies have been used:

Anti MAO-A 🡪 1:1000 (#126751, rabbit, Abcam)

Anti MAO-B 🡪 1:1000 (#M1821, rabbit, Sigma Aldrich)

Anti GAPDH 🡪 1:1000 (#51332, mouse, Cell Signaling)

Anti MYHC6 🡪 1:1000 (#ab50967, mouse, Abcam)

Anti MYHC7 🡪 1:1000 (#BA-D5, mouse, DSHB)

Anti GATA4 🡪 1:1000 (#36966, rabbit, Cell Signaling)

Anti LC3 🡪 1:1000 (#2775, rabbit, Cell Signaling)

Anti p62 🡪 1:1000 (#P0067, rabbit, Sigma Aldrich)

Anti Actin 🡪 1:1000 (#56459, mouse, Santa Cruz Biotechnology)

Anti p-AKT (S473) 🡪 1:1000 (#4060, rabbit, Cell Signaling)

Anti AKT pan 🡪 1:1000 (#4691, rabbit, Cell Signaling)

Anti p-GSK3α/β (S21/S9) 🡪 1:1000 (#9331, rabbit, Cell Signaling)

Anti p-GSK3β (S9) 🡪 1:1000 (#9336, rabbit, Cell Signaling)

Anti GSK3α/β 🡪 1:1000 (#5676, rabbit, Cell Signaling)

Anti GSK3β 🡪 1:1000 (#9315, rabbit, Cell Signaling)

Anti p-p38 🡪 1:1000 (#9215, rabbit, Cell Signaling)

Anti p38 🡪 1:1000 (#9212, rabbit, Cell Signaling)

Anti ATPB 🡪 1:1000 (#ab14730, mouse, Abcam)

Anti TOM20 🡪 1:1000 (#sc-11415, rabbit, Santa Cruz Biotechnology)

Anti PLN 🡪 1:1000 (# HPA026900, rabbit, Sigma Aldrich)

Anti p-PLN (S16/T17) 🡪 1:1000 (# SAB5701817), rabbit, Sigma Aldrich)

Anti mouse, m-IgGk BP-HRP 🡪 1:3000 (#sc-516102, mouse, Santa Cruz Biotechnology)

Anti rabbit, mouse anti-rabbit IgG-HRP 🡪 1:3000 (#sc2357, mouse, Santa Cruz Biotechnology)

**Quantitative real time PCR analysis (qRT-PCR)**

Total RNA was extracted using TRIzol (Invitrogen), and reverse transcription was performed using reverse SuperScript IV (Thermo Fisher Scientific). qRT-PCR was performed in Applied Biosystems Real Time PCR machine (StepOne) using Power SYBR Green PCR Master Mix (Applied Biosystems). Relative amounts of the gene analysed were calculated by the comparative ∆∆C(t) method. The following primers have been used:

1. *hHPRT1*
   1. Forward: GACCAGTCAACAGGGGACAT
   2. Reverse: CCTGACCAAGGAAAGCAAAG
2. *hTBP*
   1. Forward: GAGAGTTCTGGGATTGTACCG
   2. Reverse: ATCCTCATGATTACCGCAGC
3. *hB2M*
   1. Forward: CACCCCCACTGAAAAAGATG
   2. Reverse: ATATTAAAAAGCAAGCAAGCAGAA
4. *hGAPDH*
   1. Forward: ACCCACTCCTCCACCTTTG
   2. Reverse: CTCTTGTGCTCTTGCTGGG
5. *hNANOG*
   1. Forward: AGTCCCAAAGGCAAACAACCCACTTC
   2. Reverse: TGCTGGAGGCTGAGGTATTTCTGTCTC
6. *hMESP1*
   1. Forward: CTGCCTGAGGAGCCCAAGT
   2. Reverse: GCAGTCTGCCAAGGAACCA
7. *hGATA4*
   1. Forward: TCCAAACCAGAAAACGGAAG
   2. Reverse: AAGACCAGGCTGTTCCAAGA
8. *NKX2.5*
   1. Forward: CAAGGACCCTAGAGCCGAAAAG
   2. Reverse: TTGACCTGCGTGGACGTGAGTTTC
9. *WNT3*
   1. Forward: CGGCGCCTCTTCTAATGGAG
   2. Reverse: GCCCAGAGATGTGTACTGCT
10. *WNT3A*
    1. Forward: ATGGCCCCACTCGGATACT
    2. Reverse: GGAGGAATACTGTGGCCCAA
11. *WNT11*
    1. Forward: ATATCCGGCCTGTGAAGGAC
    2. Reverse: TGCACTGCCTGTCTTGTGTC
12. *SERCA2A*
    1. Forward: CGAACCCTTGCCACTCATCT
    2. Reverse: CAGGTTCCAGGTAGTTGCGG
13. *RYR2*
    1. Forward: CATCGAACACTCCTCTACGGA
    2. Reverse: GGACACGCTAACTAAGATGAGGT
14. *MCU*
    1. Forward: AGTTCACACTCAAGCCTATCTCT
    2. Reverse: TCAATTCCCCGATCCTCTTCTT
15. *PLN*
    1. Forward: ACCTCACTCGCTCAGCTATAA
    2. Reverse: CATCACGATGATACAGATCAGCA
16. *NCX1*
    1. Forward: TCATAGCTGATCGGTTCATGTCC
    2. Reverse: CAGTTGTCTTGGTGGTCTCTC
17. *RCAN1*
    1. Forward: GCTCCGCCAAATCCAGACAA
    2. Reverse: GCTGCGTGCAATTCATACTTTTC

**Scoring analysis of beating hiPSC-CMs**

hiPSCs were plated at a density of 3 x 10^4^ cells/well in 96-well plates and differentiated as described before. Cells were differentiated in the presence of N-2-mercaptopropionylglycine (MPG) 300 µM (Sigma-Aldrich), mitoTEMPO 10 µM (Sigma-Aldrich), pargyline 100 µM, apocynin (Sigma-Aldrich) 0.25, 0.5, 1 mM and Wnt-C59 (Cayman) 0.5, 1 and 2 µM. At day 20 of differentiation, wells containing 2 or more clusters of beating hiPSC-CMs were considered beating wells.

Every group was analysed as a percentage of beating wells normalized to the number of total wells per group.

**Pluripotency marker analysis**

hiPSCs colonies were grown in 12-well plates on 1% Geltrex matrix (Thermo Fisher Scientific) coated coverslips. hiPSCs colonies were fixed with 4% paraformaldehyde at room temperature for 15 min and subsequently washed 3 times with PBS. Cells were permeabilized with 0.5% Triton X-100 for 15 min at room temperature and blocked using 3% bovine serum albumin (BSA) for 30 min. Colonies were incubated at 4°C O/N with primary antibodies directed against NANOG (Thermo Fisher Scientific, #PA1-097, rabbit) and SSEA4 (Thermo Fisher Scientific, #MC-813-70, mouse), diluted 1:100 in blocking buffer (3% BSA). Cells were washed in PBS three times and incubated with secondary antibodies Alexa Fluor 488 conjugated anti-mouse (Thermo Fisher Scientific, 1:250) or Alexa Fluor 594 conjugated anti-rabbit (Thermo Fisher Scientific, 1:250) at room temperature for 1h in 3% BSA blocking buffer. The colonies were washed with PBS three times and coverslips mounted using ProLong Diamond Antifade Mountant with DAPI (Life Technologies). Images were collected at Zeiss LSM 700 confocal system equipped with a PlanApo 40x/1.2 oil objective at 2048x2048 pixels per image with a 100 Hz acquisition rate and analyzed using the Fiji distribution of the Java-based image processing program ImageJ [[5](#_ENREF_5)].

## **Transmission Electron Microscopy**

hiPSCs were plated in 12-multiwell plates and, in the case of hiPSC-CMs, cells were further differentiated for 20 days and then fixed with 2.5% glutaraldehyde in sodium cacodylate 0.1 M pH 7.4 for 1 hour at 4°C, and then postfixed with 1% osmium tetroxide and 1.5% potassium ferrocyanide in 0.1 M sodium cacodylate pH 7.4 for 1 hour at 4°C. Before embedding in epoxy embedding medium (Fluka), cells were dehydrated through a graded series of ethanol. Samples were stained with uranyl acetate and lead citrate and the sections were analyzed under a Tecnai-12 transmission electron microscope operating at 100 kV (Philips-FEI). Images were collected by a F114 (TVIPS) CCD camera. TEM samples preparation and image acquisition were performed by the Department of Biology (University of Padua) electron microscopy facility.

## **Mitochondrial Morphometry Analysis and ERMICC**

Mitochondrial perimeter and mito-ER contact sites were quantified using the ImageJ FIJI freehand tool line. The ER–mitochondria contact coefficient (ERMICC) was used to analyze the extent of physical interaction between mitochondria and ER because it takes into account the distance between the ER and mitochondria and also the length of the contact between membranes and the perimeter of the mitochondria involved in the interaction [[3](#_ENREF_3)].

Four independent experiments were performed and approximately 80 fields of view per experiment were quantified.

**Supplementary Figures and Figure Legends
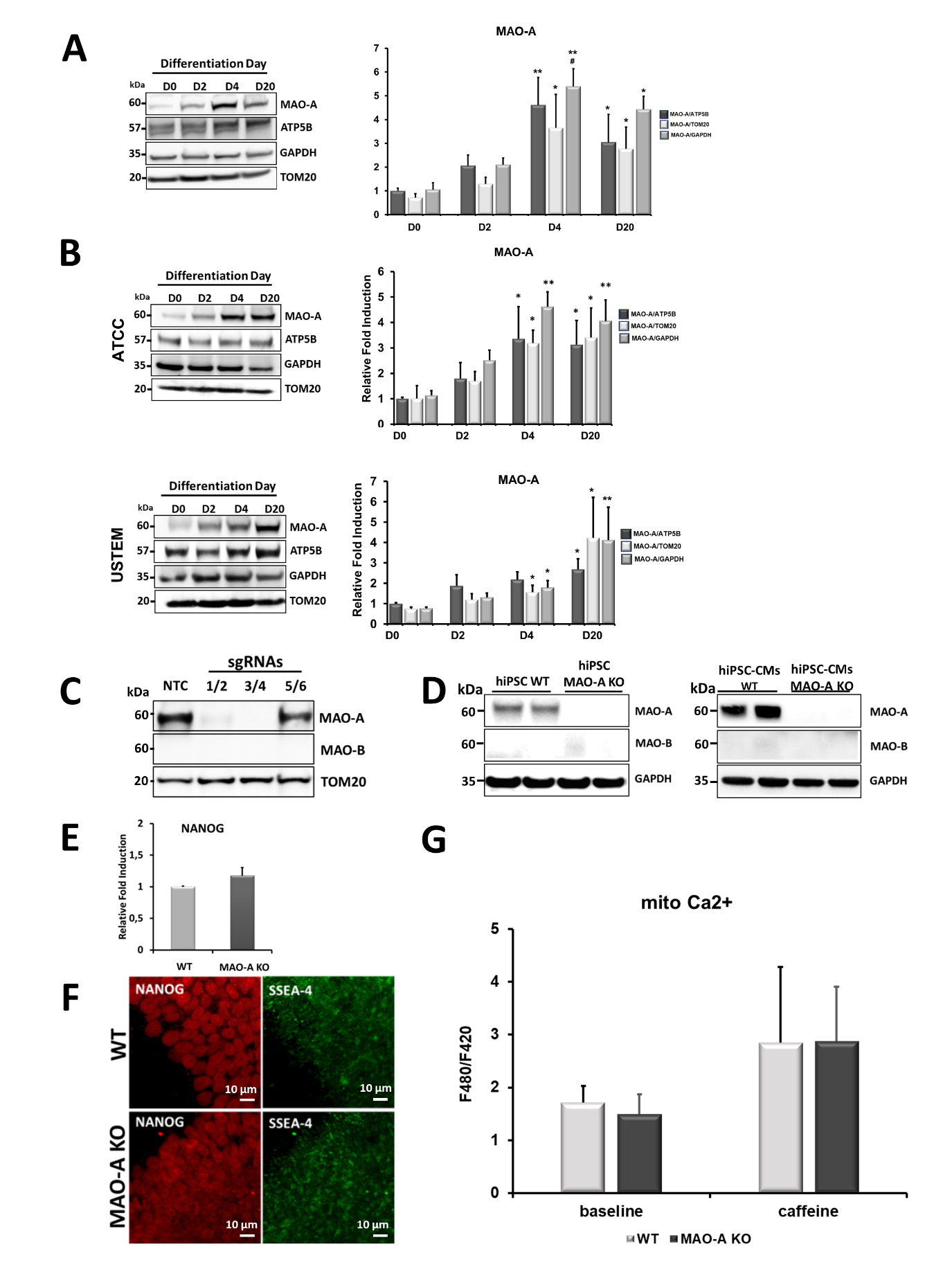
**

**Supplementary Figure 1. Analysis of MAO-A expression during cardiac lineage commitment and characterization of MAO-A KO hiPSCs**. **A-B** MAO-A protein expression during different stages of cardiomyocyte differentiation in SCVI15, ATCC and USTEM hiPSCs, respectively. Densitometry analysis is shown on the right. MAO-A expression at day 0 (D0) was arbitrarily considered as a unit. MAO-A values were normalized to ATP5B, TOM20 or GAPDH. *p<0.05 vs D0, **p<0.005 vs D0, #p<0.05 vs D2 by one-way ANOVA, Dunn’s post hoc pairwise comparison. **C** MAO-A and MAO-B protein expression in HEK293T cells infected with virus particles carrying three different sgRNAs against MAO-A (sgRNA1/2, sgRNA3/4, sgRNA5/6). Expression level of TOM20 protein was used as loading control. **D** MAO-A and MAO-B protein expression levels in WT and MAO-A KO hiPSCs and hiPSC-CMs. Expression level of GAPDH protein was used as loading control. **E** *NANOG* mRNA expression levels in WT and MAO-A KO hiPSCs. WT values were arbitrarily considered as a unit. Values were normalized to *GAPDH*. **F** Representative images for NANOG (red) and SSEA-4 (green) immunofluorescent staining in WT and MAO-A KO hiPSCs. Scale bar 10 µm. **G** Quantification of mitochondrial Ca^2+^ levels at baseline and following caffeine stimulation.

All experiments were performed at least three times using three different preparations. Results are expressed as mean ± S.E.M.

**
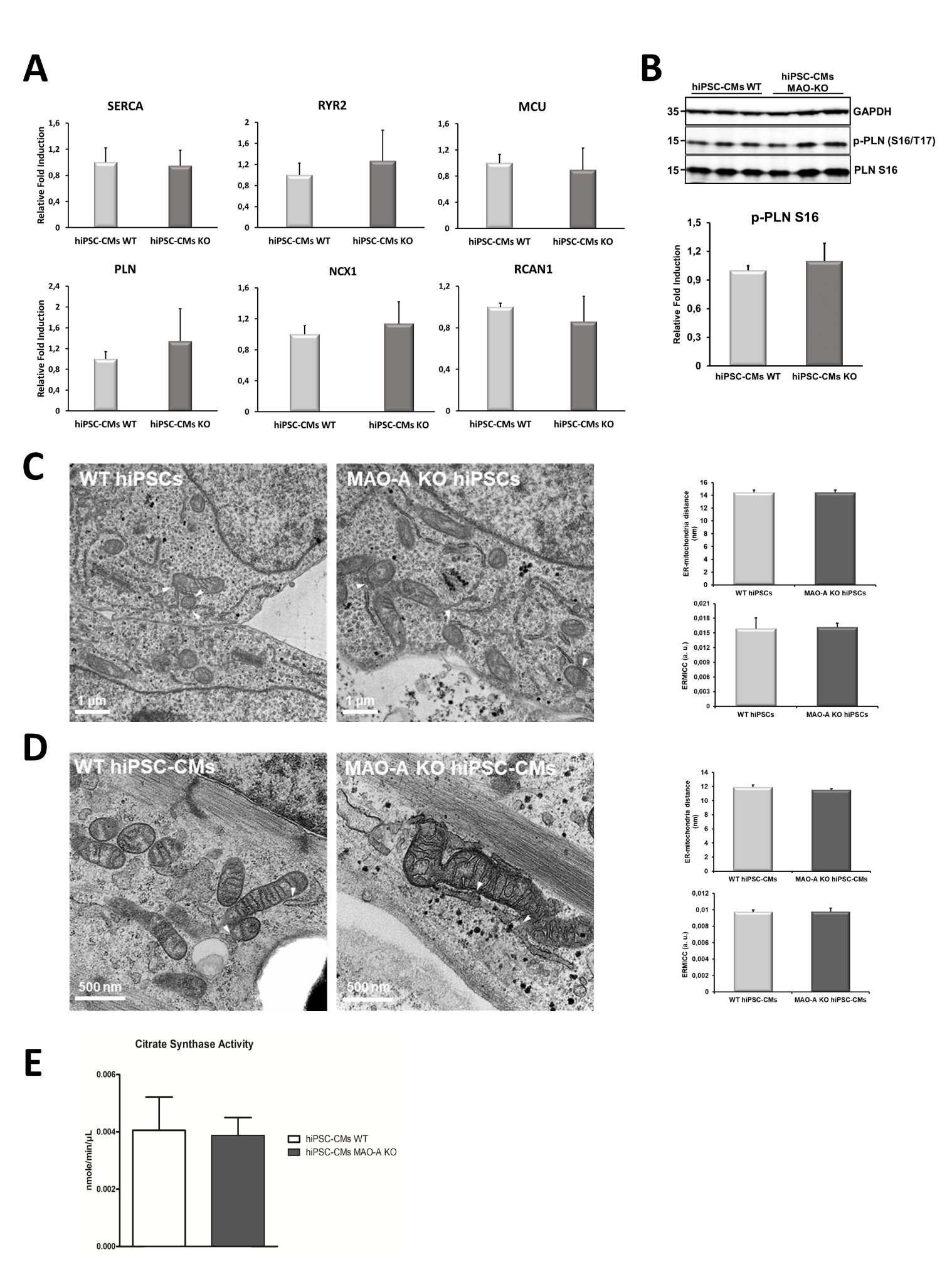
**

**Supplementary Figure 2**. **Effect of MAO-A deletion on calcium handling proteins and analysis of mitochondria-ER/SR contact sites. A** Analysis of gene expression level of Ca^2+^ handling proteins in hiPSC-CMs. Values expressed in hiPSC-CMs WT cells were arbitrarily considered as a unit. Data were normalized to GAPDH and analyzed by two-tailed Student’s t test. **B** Western blotting analysis of PLN in hiPSC-CMs WT and MAO-A KO cells. The phosphorylation level of PLN (S16/T17) was normalized to total protein levels. Data were analyzed by two-tailed Student’s t test. All experiments were performed at least three times using three different preparations. Results are expressed as mean ± S.E.M. **C-D** Representative EM images of hiPSCs and hiPSC-CMs of the indicated genotype. Densitometry analysis is shown in the right panel. Mean ± SEM of ERMICC was calculated from four independent experiments. Data were analyzed by two-tailed Student’s t test. **E** Citrate synthase activity of WT and MAO-A KO hiPSC-CMs. Experiments were statistically analyzed by two-tailed Student’s t test. All experiments were performed at least three times using three different preparations. Results are expressed as mean ± S.E.M.

**
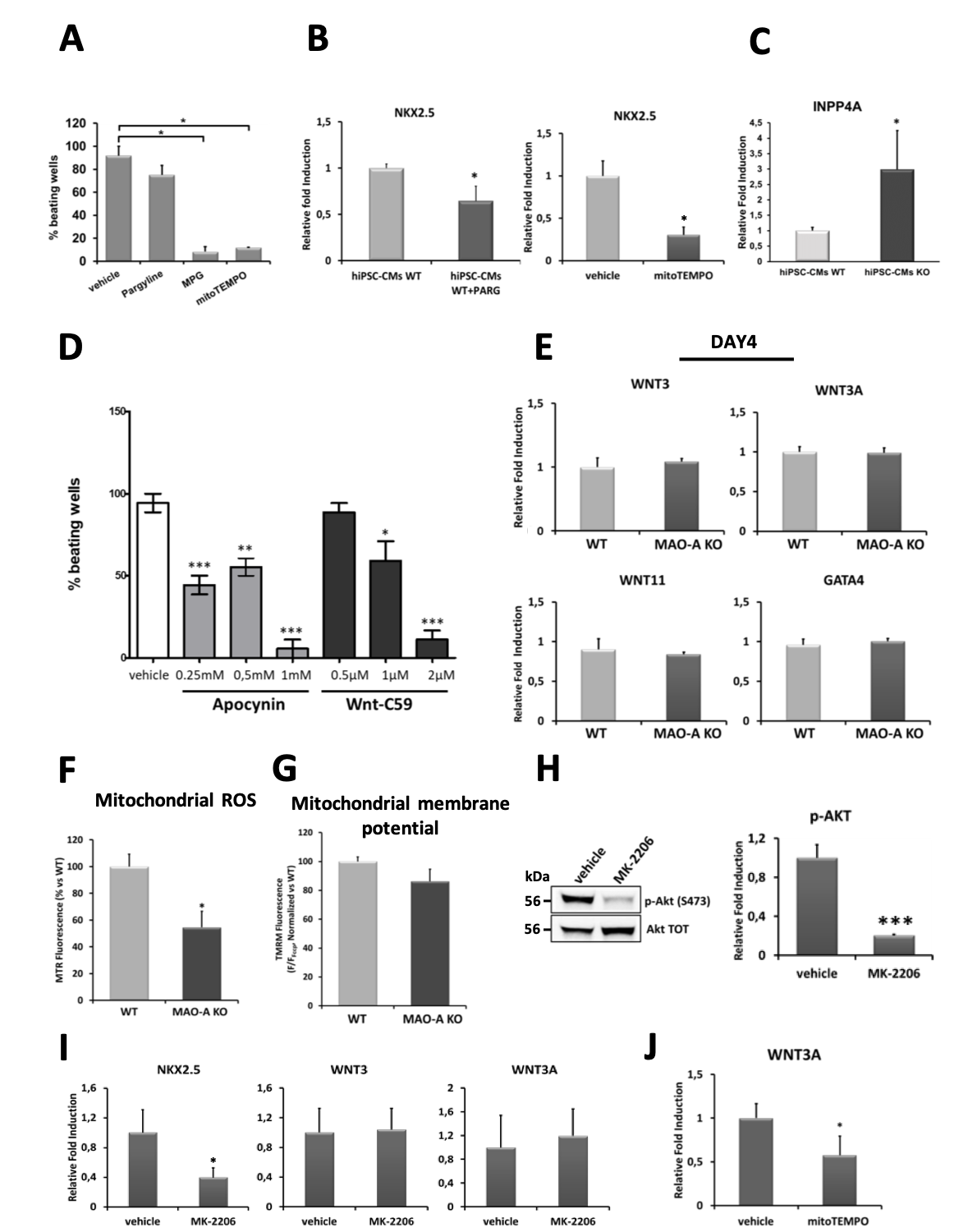
**

**Supplementary Figure 3**. **Effect of ROS scavengers, NOX and WNT inhibition and MAO-A deletion on cardiomyocyte differentiation.** **A** After 20 days of differentiation in the presence of indicated compounds, wells containing 2 or more beating foci were considered as beating wells. Experiments were normalized and expressed as % vs vehicle. *p<0.001 by one-way ANOVA with *post hoc* Tukey’s multiple comparison test. Experiments were performed three times and 12 wells per condition were scored in every experiment. **B** *NKX2.5* mRNA expression levels in pargyline and mitoTEMPO-treated cells on day 6 of differentiation. Vehicle values were arbitrarily considered as a unit. Values were normalized to *GAPDH*. *p<0.01 by two-tailed Student’s t test. **C** *INPP4A* mRNA expression levels in WT and MAO-A hiPSC-CMs. WT values were arbitrarily considered as a unit. Values were normalized to *GAPDH*. *p<0.01 by two-tailed Student’s t test. **D** Analysis of the effect of apocynin and Wnt-C59 treatment on cardiomyocyte differentiation. Wells containing 2 or more beating foci were considered as beating wells. Experiments were normalized and expressed as % vs vehicle. *p<0.05, **p<0.01, ***p<0.001 by one-way ANOVA with *post hoc* Tukey’s multiple comparison test. Experiments were performed three times and 6 wells per condition were scored in every experiment. **E** *WNT3, WNT3A, WNT11*, and *GATA4* mRNA expression levels in WT and MAO-A KO cells at day 4 of differentiation. For each gene, WT values were arbitrarily considered as a unit. Values were normalized to *GAPDH* and analyzed by two-tailed Student’s t test. **F** Mitochondrial ROS levels in WT and MAO-A KO cells at day 6 of cardiomyocyte differentiation. Values were normalized and expressed as % vs WT D6. *p<0.001 by two-tailed Student’s t test. At least 100 cells were analyzed per condition in each experiment. **G** Mitochondrial membrane potential in WT and MAO-A KO cells at day 6 of cardiomyocyte differentiation. Results are expressed as F/F_FCCP_, normalized to WT D6 and statistically analyzed by two-tailed Student’s t test. At least 30 cells were analyzed per condition in each experiment. **H** Representative western blot showing reduction in AKT phosphorylation after treatment with MK-2206 for 4 days during mesoderm/cardiac specification. Densitometric analysis is shown on the right. For each group vehicle values were arbitrarily considered as a unit. Phosphorylation levels were normalized vs total protein levels. *p<0.001 by two-tailed Student’s t test. **I** *NKX2.5, WNT3* and *WNT3A* mRNA expression levels at day 6 of differentiation following AKT inhibition with MK-2206. Vehicle values were arbitrarily considered as a unit. Values were normalized to *GAPDH*. *p<0.001 by two-tailed Student’s t test. **J** *WNT3A* mRNA expression levels at day 6 of differentiation following treatment of WT cells with mitoTEMPO. Vehicle values were arbitrarily considered as a unit. Values were normalized to *GAPDH*. *p<0.05 by two-tailed Student’s t test.

All experiments were performed at least three times using three different preparations. Results are expressed as mean ± S.E.M.

**
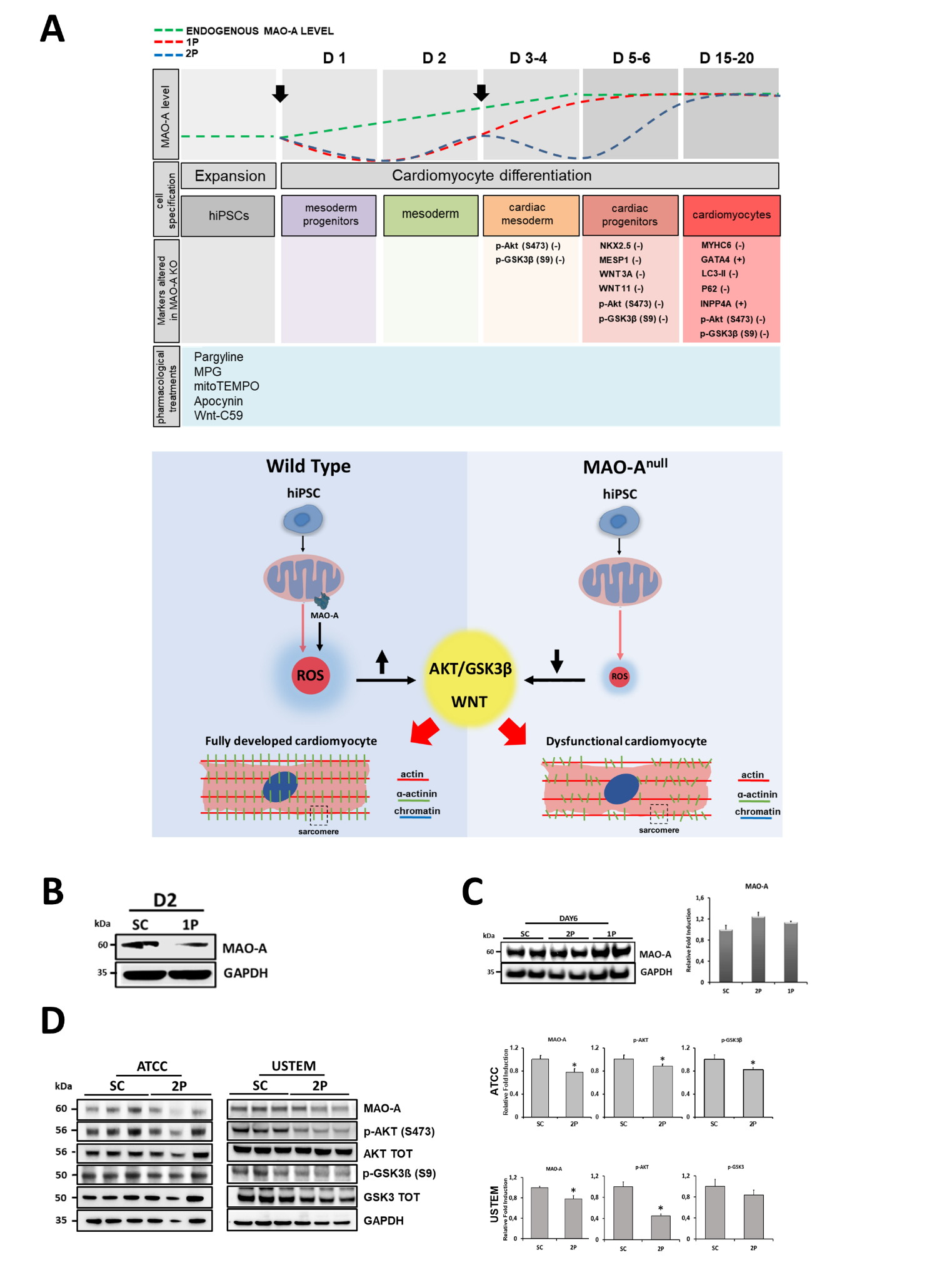
**

**Supplementary Figure 4. MAO-A expression and siRNA treatment during cardiac differentiation.** **A** Cells were transfected with siRNA at indicated time points (arrows). The green line represents endogenous MAO-A protein levels during cardiac differentiation. The red line represents MAO-A protein level upon 1 pulse of siRNA (1P). The blue line represents MAO-A protein level upon 2 pulses of siRNA (2P). The lower panels include a summarizing scheme of approaches used (i.e. genetic vs pharmacological) as well as signaling pathways involved downstream of MAO-A. **B** MAO-A protein expression after 2 days of scramble (SC) or 1 pulse of MAO-A siRNA treatment. **C** MAO-A expression levels at day 6 of differentiation following treatment with scramble (SC) RNA, 1 pulse or 2 pulses of siRNA against MAO-A (1P and 2P, respectively). Densitometry analysis is shown in the right panel. SC values were arbitrarily considered as a unit and MAO-A levels were normalized to GAPDH. Data were analyzed by one-way ANOVA with *post hoc* Tukey’s multiple comparison test. **D** MAO-A protein levels and AKT/GSK3β phosphorylation following treatment with scramble (SC) RNA or 2 pulses (2P) of siRNA against MAO-A in ATCC and USTEM cells. Densitometry analyses for MAO-A expression levels, and AKT and GSK3β phosphorylation on day 4 are shown on the right. For each group, SC values were arbitrarily considered as a unit. Phosphorylation levels were normalized to total protein levels. *p<0.05 by two-tailed Student’s t test. All experiments were performed at least three times using three different preparations. Results are expressed as mean ± S.E.M.


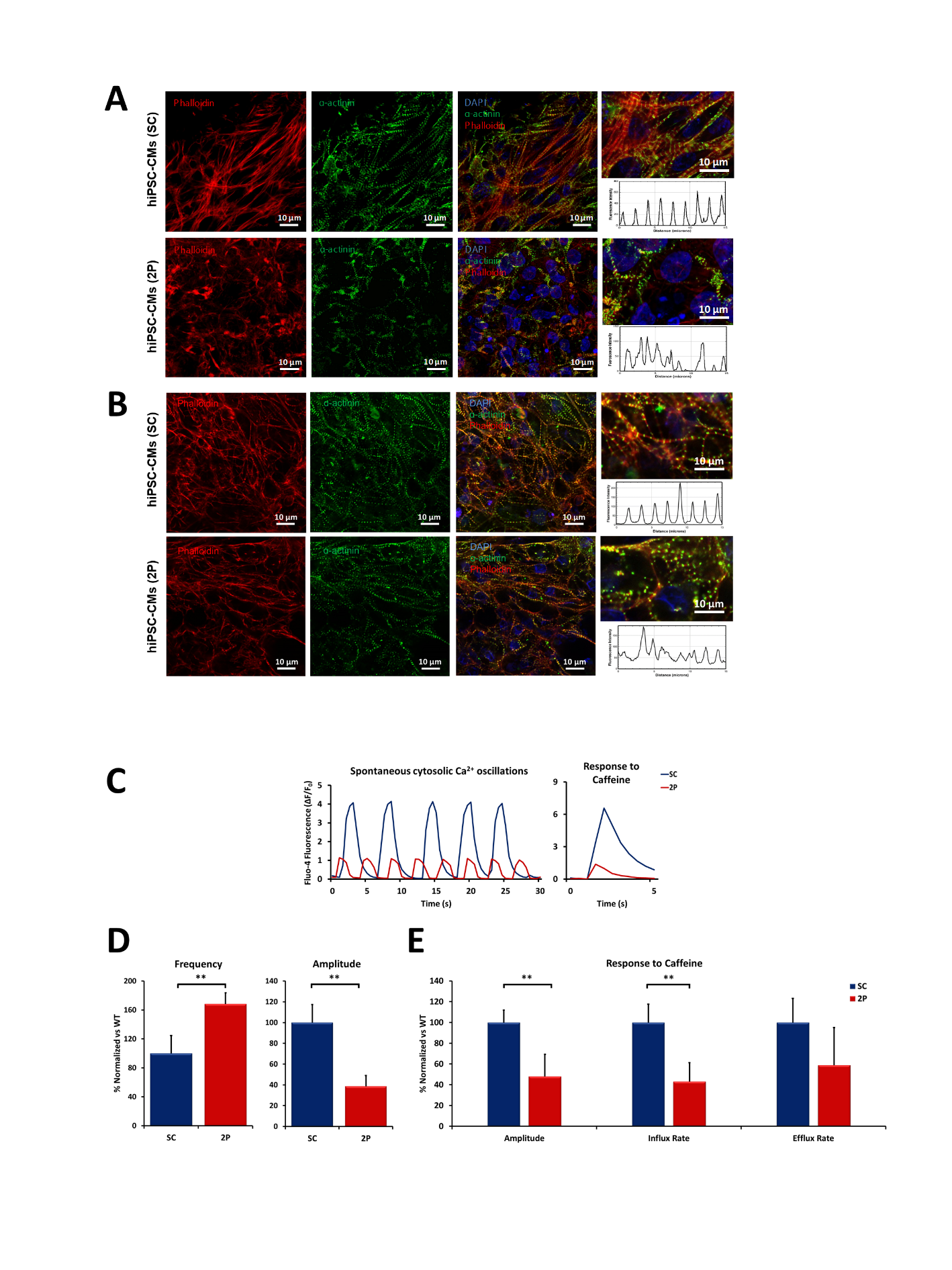
**Supplementary Figure 5. Effect of MAO-A siRNA on hiPSC-CMs structure and Ca^2+^ homeostasis.** **A-B** α-sarcomeric actinin (green) immunofluorescent labeling in cells obtained from healthy donors (ATCC and USTEM, respectively) treated with scramble (SC) RNA or 2 pulses (2P) of MAO-A siRNA. Phalloidin is shown in red, while nuclei were stained with DAPI (blue). The patterning of α-sarcomeric actinin fluorescence intensity is plotted on the right, denoting sarcomere organization within the cell. Approximately 20/30 cells were analyzed in each experiment. Scale bar 10 µm. **C** Representative traces of spontaneous cytosolic Ca^2+^ oscillations (left panel) and cytosolic Ca^2+^ peak induced by caffeine stimulation (right panel) in ATCC derived hiPSC-CMs treated with scramble (SC) RNA, and 2 pulses (2P) of siRNA against MAO-A. **D** Quantification of spontaneous cytosolic Ca^2+^ oscillations frequency and peak amplitude. **p<0.001 by two-tailed Student’s t test. All experiments were performed at least three times using three different preparations. Five regions of interest (ROIs) were selected in each field of view and at least three different fields of view were analyzed in each experiment. **E** Quantification of caffeine-induced cytosolic Ca^2+^ peak in terms of peak amplitude, influx and efflux rate. **p<0.01 by two-tailed Student’s t test. All experiments were performed at least three times using three different preparations.

All experiments were performed at least three times using three different preparations. Results are expressed as mean ± S.E.M.

**References**

1. Drawnel FM, Boccardo S, Prummer M, Delobel F, Graff A, Weber M, Gerard R, Badi L, Kam-Thong T, Bu L, Jiang X, Hoflack JC, Kiialainen A, Jeworutzki E, Aoyama N, Carlson C, Burcin M, Gromo G, Boehringer M, Stahlberg H, Hall BJ, Magnone MC, Kolaja K, Chien KR, Bailly J, Iacone R (2014) Disease modeling and phenotypic drug screening for diabetic cardiomyopathy using human induced pluripotent stem cells. Cell Rep 9:810-821. https://doi.org/:10.1016/j.celrep.2014.09.055

2. Kaludercic N, Carpi A, Nagayama T, Sivakumaran V, Zhu G, Lai EW, Bedja D, De Mario A, Chen K, Gabrielson KL, Lindsey ML, Pacak K, Takimoto E, Shih JC, Kass DA, Di Lisa F, Paolocci N (2014) Monoamine oxidase B prompts mitochondrial and cardiac dysfunction in pressure overloaded hearts. Antioxid Redox Signal 20:267-280. https://doi.org/:10.1089/ars.2012.4616

3. Naon D, Zaninello M, Giacomello M, Varanita T, Grespi F, Lakshminaranayan S, Serafini A, Semenzato M, Herkenne S, Hernandez-Alvarez MI, Zorzano A, De Stefani D, Dorn GW, 2nd, Scorrano L (2016) Critical reappraisal confirms that Mitofusin 2 is an endoplasmic reticulum-mitochondria tether. Proc Natl Acad Sci U S A 113:11249-11254. https://doi.org/:10.1073/pnas.1606786113

4. Sanjana NE, Shalem O, Zhang F (2014) Improved vectors and genome-wide libraries for CRISPR screening. Nat Methods 11:783-784. https://doi.org/:10.1038/nmeth.3047

5. Schindelin J, Arganda-Carreras I, Frise E, Kaynig V, Longair M, Pietzsch T, Preibisch S, Rueden C, Saalfeld S, Schmid B, Tinevez JY, White DJ, Hartenstein V, Eliceiri K, Tomancak P, Cardona A (2012) Fiji: an open-source platform for biological-image analysis. Nat Methods 9:676-682. https://doi.org/:10.1038/nmeth.2019

6. Shalem O, Sanjana NE, Hartenian E, Shi X, Scott DA, Mikkelson T, Heckl D, Ebert BL, Root DE, Doench JG, Zhang F (2014) Genome-scale CRISPR-Cas9 knockout screening in human cells. Science 343:84-87. https://doi.org/:10.1126/science.1247005

7. Shearer RF, Saunders DN (2015) Experimental design for stable genetic manipulation in mammalian cell lines: lentivirus and alternatives. Genes Cells 20:1-10. https://doi.org/:10.1111/gtc.12183

8. Tohyama S, Hattori F, Sano M, Hishiki T, Nagahata Y, Matsuura T, Hashimoto H, Suzuki T, Yamashita H, Satoh Y, Egashira T, Seki T, Muraoka N, Yamakawa H, Ohgino Y, Tanaka T, Yoichi M, Yuasa S, Murata M, Suematsu M, Fukuda K (2013) Distinct metabolic flow enables large-scale purification of mouse and human pluripotent stem cell-derived cardiomyocytes. Cell Stem Cell 12:127-137. https://doi.org/:10.1016/j.stem.2012.09.013
